# A Regulatory Loop Involving PAX6, MITF, and WNT Signaling Controls Retinal Pigment Epithelium Development

**DOI:** 10.1371/journal.pgen.1002757

**Published:** 2012-07-05

**Authors:** Kapil Bharti, Melanie Gasper, Jingxing Ou, Martha Brucato, Katharina Clore-Gronenborn, James Pickel, Heinz Arnheiter

**Affiliations:** 1 Mammalian Development Section, National Institute of Neurological Disorders and Stroke, National Institutes of Health, Bethesda, Maryland, United States of America; 2 Transgenic Core Facility, National Institute of Mental Health, National Institutes of Health, Bethesda, Maryland, United States of America; Medical Research Council Human Genetics Unit, United Kingdom

## Abstract

The separation of the optic neuroepithelium into future retina and retinal pigment epithelium (RPE) is a critical event in early eye development in vertebrates. Here we show in mice that the transcription factor PAX6, well-known for its retina-promoting activity, also plays a crucial role in early pigment epithelium development. This role is seen, however, only in a background genetically sensitized by mutations in the pigment cell transcription factor MITF. In fact, a reduction in *Pax6* gene dose exacerbates the RPE-to-retina transdifferentiation seen in embryos homozygous for an *Mitf* null allele, and it induces such a transdifferentiation in embryos that are either heterozygous for the *Mitf* null allele or homozygous for an RPE–specific hypomorphic *Mitf* allele generated by targeted mutation. Conversely, an increase in *Pax6* gene dose interferes with transdifferentiation even in homozygous *Mitf* null embryos. Gene expression analyses show that, together with MITF or its paralog TFEC, PAX6 suppresses the expression of *Fgf15* and *Dkk3*. Explant culture experiments indicate that a combination of FGF and DKK3 promote retina formation by inhibiting canonical WNT signaling and stimulating the expression of retinogenic genes, including *Six6* and *Vsx2*. Our results demonstrate that in conjunction with *Mitf/Tfec Pax6* acts as an anti-retinogenic factor, whereas in conjunction with retinogenic genes it acts as a pro-retinogenic factor. The results suggest that careful manipulation of the *Pax6* regulatory circuit may facilitate the generation of retinal and pigment epithelium cells from embryonic or induced pluripotent stem cells.

## Author Summary

The retinal pigment epithelium or RPE in the back of the eye is critical for the normal function of the retina, and its abnormalities can lead to retinal disorders such as adult-onset macular degeneration. Insights into the pathogenesis of such disorders, and potential therapies, may come from using RPE cells generated in vitro from induced pluripotent stem cells. To obtain authentic RPE cells in vitro, we need to thoroughly understand the normal process of their development in vivo. Here we find that the potent retina-inducing transcription factor PAX6 plays a critical anti-retinogenic role in the RPE of mice. But how can PAX6 be pro-retinogenic in the retina and anti-retinogenic in the RPE? To address this question, we used gene expression studies and combined them with chromatin immunoprecipitation assays, which analyze the interaction of transcription factors with chromatin in vivo. Our findings show that, in the RPE, PAX6 cooperates with either one (or both) of two related RPE transcription factors, MITF and TFEC, to suppress extracellular signals that in the normal retina induce a signaling cascade promoting retina formation. Hence, this study provides mechanistic insights into RPE development that may become important for the efficient generation of retina and RPE from induced pluripotent stem cells.

## Introduction

The vertebrate retinal pigment epithelium or RPE is a monolayer of melanin-containing cells that are intimately juxtaposed to the photoreceptor layer of the retina where they function as light screen and provide metabolic support for the retina's photoreceptors. Like the retina, the RPE is developmentally derived from the optic neuroepithelium and can be converted experimentally to retina, usually only during early development but in some species, such as newts, even in adults [1]–[3].

The differentiation of the optic neuroepithelium into precursors for retina and RPE is controlled by numerous growth factors and transcription factors. Among them is the *microphthalmia*-associated transcription factor MITF, which is a basic-helix-loop-helix-leucine zipper protein that together with the closely related proteins TFE3, TFEB and TFEC forms the Mi-T subfamily of MYC-related transcription factors [4], [5]. During mouse eye development, when the budding optic vesicle has not yet touched the surface ectoderm, low levels of MITF are found throughout the vesicle's epithelium. *Mitf* then is downregulated in the distal part of the vesicle, the future retina, in a pathway that involves FGF signaling and the paired-like homeodomain transcription factor VSX2 [6], [7], and it is upregulated in the proximal part, the future RPE, in a pathway that, based on work mostly in the chick, involves bone morphogenetic proteins (BMPs), several transforming growth factor-ßs including ACTIVIN, and WNT signaling [8]–[12]. If *Mitf* is missing, an optic cup still forms and retina development continues, but the domain that normally becomes RPE hyperproliferates, remains unpigmented, and in its dorsal part turns into a second, fully laminated retina in a process commonly referred to as “transdifferentiation” [7], [13]. Conversely, if *Mitf* fails to be downregulated in the distal optic vesicle, the domain that normally becomes retina hypo-proliferates and turns into a pigmented monolayer resembling the RPE [6], [7], [14].

A second transcription factor initially expressed in both distal and proximal optic vesicle is the paired domain protein PAX6. Unlike MITF, PAX6 initially remains high in both future retina and RPE but later fades away in the RPE [14]–[16]. PAX6 has widespread roles in vertebrate eye development, both in structures derived from the surface ectoderm, such as cornea and lens, and in those derived from the neuroepithelium, such as iris, ciliary body, and retina [17]. Indeed, if in mice *Pax6* is missing entirely, optic vesicles fail to form properly and eye development is aborted [18]. Conditional ablation specifically in the neuroretina allows for neuroretinal development to proceed but retinal progenitor cells can only mature into amacrine interneurons instead of the many different cell types of a normal retina [19]. In the RPE, however, the roles of *Pax6* are less clear as an early RPE-specific ablation is not yet available and overexpression has no major effects on RPE development [20], [21]. Nevertheless, studies involving chimeras between *Pax6* wild-type and mutant mouse embryos showed that *Pax6*-deficient cells can contribute to the RPE although their differentiation (pigmentation) is impaired [22].

There are a number of previous studies that have suggested cross-regulations between *Mitf* and *Pax6*. Biochemical studies, for instance, have indicated that PAX6 and MITF interact at the protein level, and that this interaction results in a mutual inactivation of both proteins [23]. In vitro, PAX6 stimulates at least one of the multiple *Mitf* promoters, the 5′ most human A-MITF promoter, and the combined absence of PAX6 and PAX2, though not the absence of PAX6 alone, abolishes MITF expression in the optic neuroepithelium of mouse embryos as visualized by immunofluorescence [24]. Furthermore, a reduction of functional MITF in the RPE leads to an increase in PAX6 expression, most prominently in the transdifferentiating but also in the non-transdifferentiating portion [7].

Given the above findings, we reasoned that a decrease in *Pax6* gene dose would alleviate RPE transdifferentiation in *Mitf* mutants, and upregulation further expand this transdifferentiation. Hence, we crossed mice carrying mutant *Mitf* alleles either with mice carrying the *Pax6^Sey-Neu^* allele, which represents a functional null allele [18], or mice overexpressing human *PAX6* from a yeast artificial chromosome (YAC) transgene [20], [21]. Contrary to expectation, however, we find that a reduction of *Pax6* enhances RPE transdifferentiation in *Mitf* mutants and overexpression suppresses it. In fact, overexpression of *PAX6* increases the expression of the RPE-specific *Mitf* paralog *Tfec* which helps to compensate for the loss of the anti-proliferative, though not the pigmentary, function of *Mitf*. By gene expression profiling, we identified two major targets of the *Pax6/Mitf/Tfec* pathway that act extracellularly: *Fgf15*, a member of the FGF family of ligands that serves as a positive regulator of retinal development, and *Dkk3*, which together with FGFs inhibits RPE-promoting WNT signaling. Hence, our results show that despite the common neuroepithelial origin of retina and RPE, the two tissues differ in the way they utilize the transcription factor PAX6. These findings may shed light on the evolution of *Pax6* as a major regulator of eye development and may have implications for designing optimal methods to obtain RPE or retinal cells for cell-therapeutic purposes from embryonic or induced pluripotent stem cells.

## Results

### *Pax6* Gene Dose Regulates RPE Transdifferentiation in *Mitf* Mutants

As seen previously [7], [15], [16] and documented in Figure S1, PAX6 and MITF protein are co-expressed in the developing RPE of mice, suggesting that the two genes interact. In fact, although the lack of *Pax6* does not alter the onset of *Mitf* expression in the optic neuroepithelium [24], the lack of *Mitf* results in increased *Pax6* expression in the RPE [7], [14], prompting us to test whether manipulating *Pax6* gene dose in embryos lacking *Mitf* would alter the development of the RPE.

**Figure 1:**
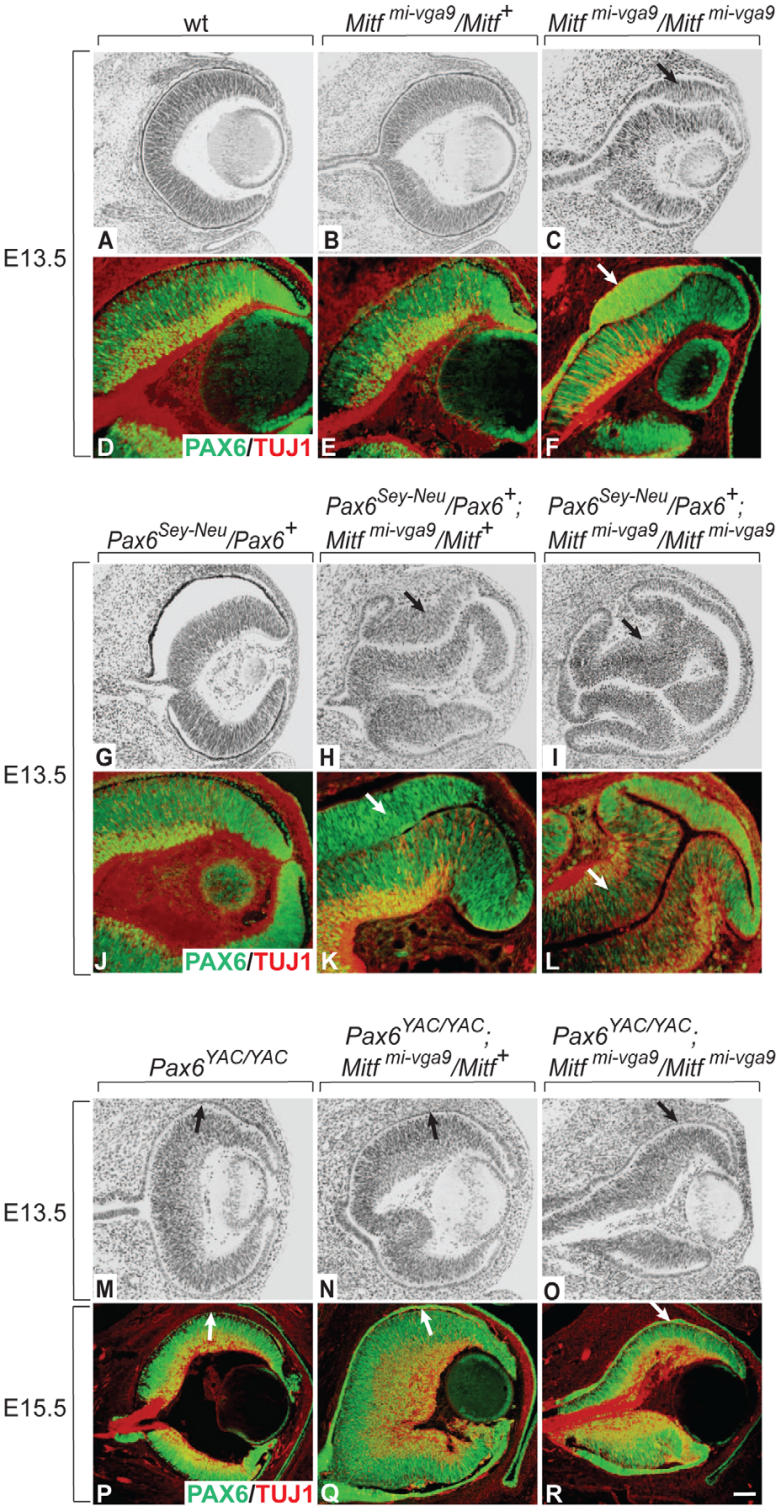
Gene dose of *Pax6* regulates dorsal RPE development in an *Mitf* mutant–lacking MITF protein. Cryostat sections of eyes of the indicated genotypes and developmental time points were stained with H&E (A–C,G–I,M–O) or labeled for the indicated markers (D–F,J–L,P–R). Note relatively mild RPE thickening in *Mitf^mi-vga9^/Mitf^mi-vga9^* (C,F). In contrast, in the presence of one copy of the *Pax6^Sey-Neu^* allele, there is a massive RPE thickening (I,L) as well as staining for the neuronal marker TUJ1 (L). In the presence of the *Pax6* YAC transgene (O,R), however, no such RPE abnormalities are seen. Arrows point to the thickened RPE (C,F,H,K,I,L) or to the corresponding monolayer RPE in (M–R). Scale bar (A–C,G–I,M–O): 115 µm; (D–F, J–L): 90 µm; (P–R): 60 µm.

As shown in Figure 1A–1F, the future RPE of embryos homozygous for the *Mitf* null allele *Mitf^mi-vga9^* showed dorsal thickening and PAX6 upregulation at E13.5 while heterozygotes showed no corresponding abnormalities (for gene structures and alleles used in this study, see Table S1). When *Mitf^mi-vga9^* heterozygous embryos were also heterozygous for the non-functional *Pax6^Sey-Neu^* allele, however, they displayed a dorsal RPE thickening reminiscent of that found in *Mitf^mi-vga9^* homozygotes (compare Figure 1H, 1K with Figure 1C, 1F). Moreover, when *Mitf^mi-vga9^* homozygotes were heterozygous for *Pax6^Sey-Neu^*, they displayed a massive enlargement of the RPE domain, which in its dorso-proximal part showed a morphology and expression pattern of the neuronal marker TUJ1 similar to those of the adjacent normal retina (Figure 1I, 1L). In contrast, when *PAX6* was overexpressed from a homozygous human *PAX6* YAC transgene (line Pax77; [21], there was no RPE thickening, not even in *Mitf^mi-vga9^* homozygotes, and this phenotypic correction persisted even after birth (Figure 1M–1R). Similar observations were made at E11.5 (Figure S2A–S2F). That the thickening of the RPE indeed resulted from cellular hyperproliferation is based on the observation that the percentage of phosphohistone H3-positive cells was significantly increased in RPEs with dorsal thickening (Figure S2G–S2K) while no changes in cell death were found (not shown). These results indicate that in the RPE domain, *Pax6* shares with *Mitf* an RPE-promoting function, in contrast to the retina domain where *Pax6* is known to promote retina development [19]. Furthermore, because the *Pax6*-dependent RPE alterations were seen in the total absence of MITF protein, PAX6/MITF protein interactions as seen in vitro [23] cannot account for the observed phenotypes.

**Figure 2:**
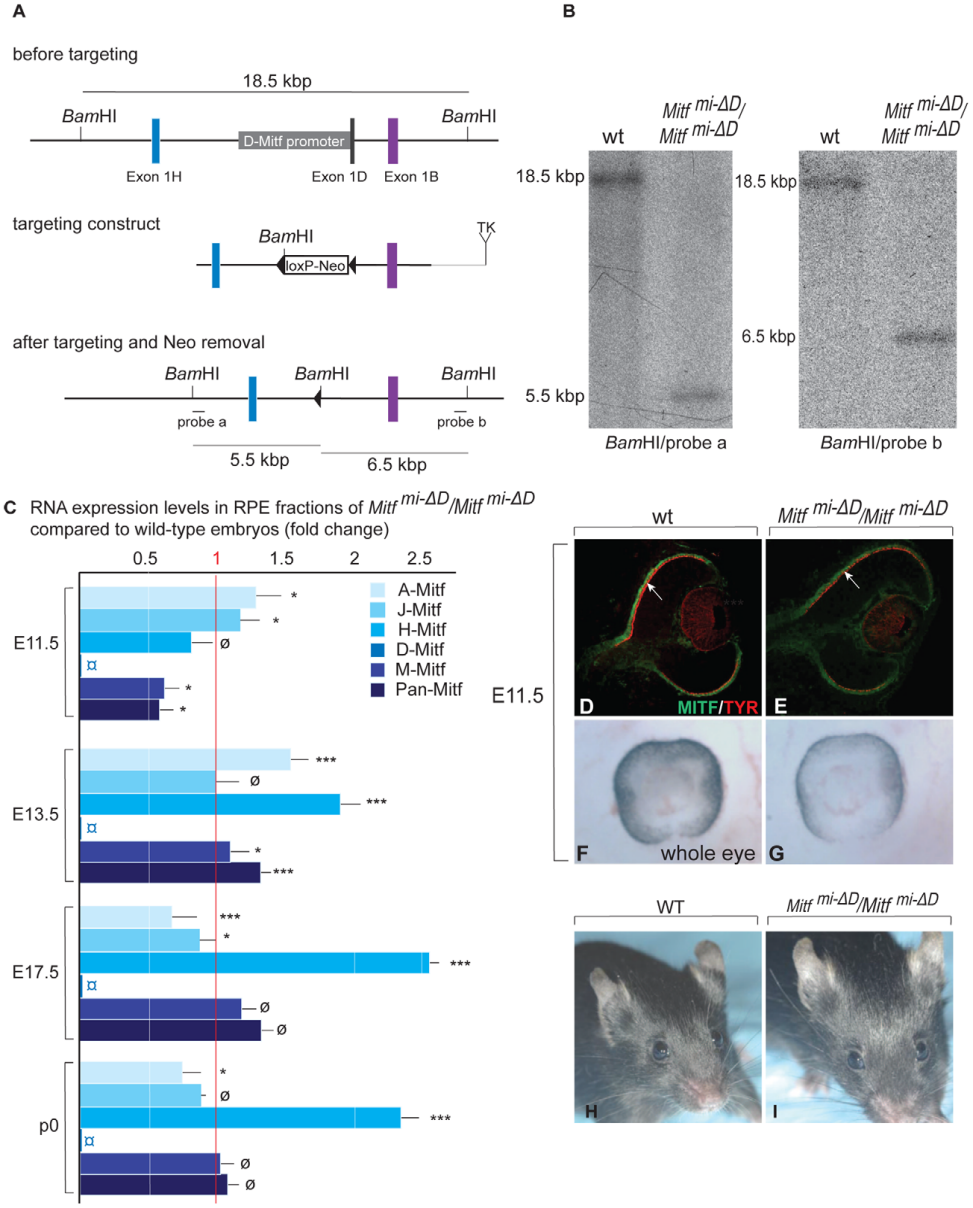
Generation and analysis of mice lacking the RPE–specific *D-isoform* of *Mitf* (*Mitf^mi-*Δ*D^/Mitf^mi-*Δ*D^*). (A) *Top*: Schematic diagrams showing a region of the *Mitf* gene containing exons 1H, 1D, and 1B; *Middle*: the targeting construct with a novel *Bam*HI restriction site and a floxed Neomycin cassette in place of 5.8 kbp of the *D-Mitf* promoter/exon 1D region; *Bottom*: *Mitf* gene portion after targeting. Probe ‘a’ recognizes a 5.5 kbp and probe ‘b’ a 6.5 kbp *Bam*HI restriction fragment after targeting while both probes recognize the same 18.5 kbp fragment before targeting (see Materials and Methods for the details on construct design). (B) Southern hybridization of *Bam*HI-restricted genomic DNA from wild-type and homozygous mutants shows the expected bands. (C) Mitf isoforms are upregulated in the RPE of *Mitf^mi-ΔD^/Mitf^mi-ΔD^* mice. Quantitative RT-PCR analysis for Mitf-isoforms from wild-type and *Mitf^mi-ΔD^/Mitf^mi-ΔD^* RPE fractions. Primers specific for Mitf isoforms A, J, H, D, and M were used to measure the respective RNAs. All values are normalized using the housekeeping gene *Usf1*. Results (mean values, S.D. and statistical significance [see Experimental Procedures] based on 3 biologically independent samples) are shown as fold change in RNA expression level in *Mitf^mi-ΔD^/Mitf^mi-ΔD^* compared to wild type. (D–G) Mice lacking the *D-isoform* of *Mitf* have reduced/delayed RPE pigmentation. Eye sections (D,E) from E11.5 wild type and *Mitf^mi-ΔD^/Mitf^mi-ΔD^* embryos show reduced MITF (green) and TYROSINASE (red) staining in the RPE (arrows in D,E). (F,G) Whole eye pictures show a mild reduction in pigmentation (F,G). (H,I) Adult *Mitf^mi-ΔD^/Mitf^mi-ΔD^* mice are indistinguishable on visual inspection from wild-type mice.

**Figure 3:**
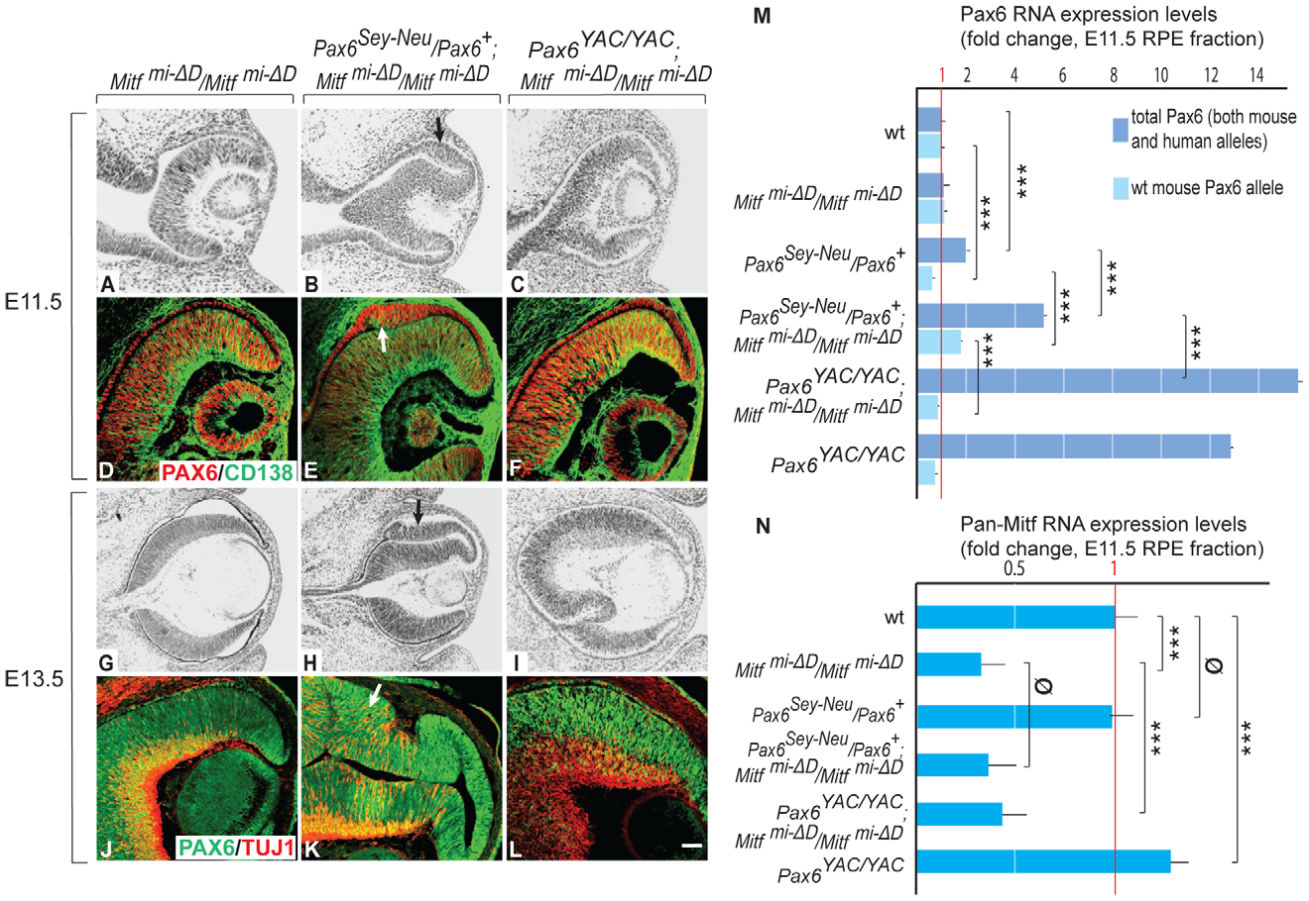
Gene dose of *Pax6* regulates dorsal RPE development in a hypomorphic *Mitf* mutant. Sectioning and labeling was performed as for Figure 1. In contrast to single *Mitf^mi-ΔD^/Mitf^mi-ΔD^* mutants, there is dorsal RPE thickening in *Pax6^Sey-Neu^/Pax6^+^; Mitf^mi-ΔD^/Mitf^mi-ΔD^* mutants (arrows in B,E,H,K). Some cells in the thickened RPE are positive for CD138, a retinal progenitor marker (arrow in E), or TUJ1, a neuronal marker (arrow in K). Scale bar (A–C,G–I): 115 µm; (D–F,J–L): 90 µm. (M,N) Expression levels of the indicated RNAs in isolated RPE fractions based on quantitative RT-PCR (fold change relative to wild type). Results represent means and S.D. obtained from 3 biologically independent samples, each representing a pool of approximately 40 RPEs. Statistical significance of the results (see Experimental Procedures) is given for multiple pairwise comparisons.

To test whether the *Pax6*-dependent RPE alterations were specific to the *Mitf^mi-vga9^* null allele, we repeated the above experiments with a newly generated RPE-specific allele of *Mitf*. We have previously found that of the many *Mitf* mRNA isoforms (see Table S1), the *D-Mitf* isoform is the only one specific to the RPE, and that this isoform contributes up to one third of total *Mitf* RNA in the RPE depending on the developmental time point [14]. Hence, by specifically eliminating the *D-Mitf* promoter/D-Mitf exon by targeted recombination, we expected to obtain mice with an RPE-specific hypomorphic allele of *Mitf*. The generation and characterization of such mice, labeled *Mitf^mi-ΔD^/Mitf^mi-ΔD^* or, for short, *D-Mitf* knock-outs, is documented in Figure 2. To test for RNA expression in their developing eyes, we used microdissected embryonic or P0 RPE or retina for quantitative RT-PCR assays as previously described [14] (Figure S3). The assays showed that *D-Mitf* RNA was completely eliminated in the RPE fraction of *D-Mitf* knock-out embryos but that other isoforms, in particular H-Mitf, were compensatorily upregulated, though not before E13.5 (Figure 2C). Consequently, total *Mitf* mRNA, quantified separately using primers common to all isoforms, was reduced only at E11.5 but not at E13.5 and thereafter (Figure 2C, see also Figure 3N). This likely explains why *D-Mitf* knock out embryos show a slight reduction in the RPE expression of the MITF target gene *Tyrosinase* and in eye pigmentation only at E11.5 (Figure 2D–2G), but normal eye development thereafter. In fact, on visual inspection, adult *D-Mitf* knock-outs look completely normal (Figure 2H, 2I).

In contrast to homozygous *D-Mitf* knock-outs carrying wild-type *Pax6* alleles (Figure 3A, 3D), homozygous *D-Mitf* knock-outs that were heterozygous for the *Pax6^Sey-Neu^* allele showed a dorsal RPE thickening at E11.5 (Figure 3B, 3E), whereby some of the cells in the thickened RPE were positive for the retinal progenitor cell marker CD138 [25] (Figure 3E, arrow). At E13.5, the dorsal thickening became prominent (Figure 3H), co-stained with TUJ1 (Figure 3K), and resembled the transdifferentiating RPE domain of *Mitf^mi-vga9^* homozygous mutants (Figure 1C, 1F). No such RPE thickening or transdifferentiation were seen in *D-Mitf* knock-outs overexpressing *PAX6* from the YAC transgene (Figure 3C, 3F, 3I, 3L). Quantitative RT-PCR measurements of total *Pax6* RNA, wild-type mouse-specific *Pax6* RNA and pan-*Mitf* RNA in the E11.5 RPE fraction of the different mutant combinations are shown in Figure 3M, 3N. Interestingly, the presence of the *Pax6^Sey-Neu^* allele led to a reduction in wild-type mouse *Pax6* RNA when *Mitf* was wild type, but to an increase when *D-Mitf* was eliminated. This increase was likely a consequence of the increase in expression of retinogenic genes including *Pax6* in the transdifferentiating portion of the RPE (Figure 3E, 3K; Figure S4). Nevertheless, the expression of just *PAX6* provided by the YAC transgene interfered with dorsal thickening or transdifferentiation. This phenomenon was likely linked to the differential regulation of the *Mitf* paralog *Tfec* (see below).

**Figure 4:**
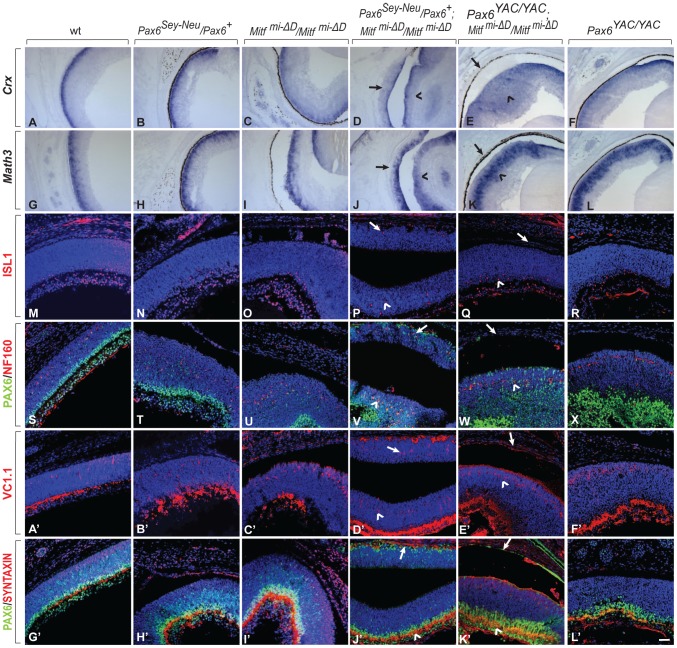
Development of a differentiated laminated retina in *Pax6^Sey-Neu^/Pax6^+^;Mitf^mi-*Δ*D^/Mitf^ mi-*Δ*D^* but not *Pax6^YAC/YAC^;Mitf ^mi-*Δ*D^/Mitf ^mi-*Δ*D^* mice. (A–L) Sections of eyes from P0 mice of the indicated genotypes were subjected to in situ hybridization for *Crx*, a photoreceptor marker (A–F) or *Math3*, an amacrine cell marker (G–L). Note that the ectopic staining is not present in the RPE of *Pax6^YAC/YAC^;Mitf ^mi-ΔD^/Mitf ^mi-ΔD^* mutants (compare arrows in D,J with E,K for ectopic staining; arrowheads mark normal retinal staining). (M–L′) Immunofluorescent labeling for the indicated markers on P0 eye sections of the indicated genotypes. ISL1 is a ganglion cell marker (M–R), as is PAX6 at this time point (S–X, G′–L′, green). NF160 marks horizontal cells (S–X, red); VC1.1 marks amacrine cells (A′–F′, red); and SYNTAXIN marks synapses (G′–L′, red). Arrows mark the transdifferentiating portions of the RPE in *Pax6^Sey-Neu^/Pax6^+^;Mitf ^mi-ΔD^/Mitf ^mi-ΔD^* mice (P,V,D′,J′) or the corresponding non-transdifferentiating portions in *Pax6^YAC/YAC^;Mitf ^mi-ΔD^/Mitf ^mi-ΔD^* mice. The normal retinas continue to express each of these markers (arrowheads in the corresponding figures). Scale bar (A–L): 115 µm; (M–X, A′–L′): 90 µm.

As documented in detail in Figure 4 and Figures S4, S5, S6, S7, the abnormal hyperproliferation of the dorsal RPE was associated with the expression of specific retinal markers and a concomitant downregulation of RPE markers (Figure S4). The abnormal RPE re-specification seems to begin in *Pax6/Mitf* double mutants already at E10.0–E10.25, just when the RPE and retina become distinct domains. As shown in Figure S5, *Six3*, which is normally expressed in the future RPE domain at E9.5 but downregulated when the cells become committed at around E10.0, continues to be expressed at low levels in this domain in both *Pax6^Sey-Neu^*/*Pax6* ^+^; *Mitf^mi-ΔD^/Mitf^mi-ΔD^* and *Pax6^Sey-Neu^*/*Pax6* ^+^; *Mitf^mi-vga9^/Mitf^mi-vga9^* mutants. Nevertheless, the retina-specific marker *Vsx2* starts to be expressed at this early time point only in the RPE domain of *Pax6^Sey-Neu^*/*Pax6* ^+^;*Mitf^mi-vga9^/Mitf^mi-vga9^* mutants and not in that of *Pax6^Sey-Neu^*/*Pax6* ^+^;*^mi-ΔD^/Mitf^mi-ΔD^*. Hence, the assessment of whether in the double mutants an RPE fate is never initiated or is first initiated and then reversed depends on the retinal markers used and the particular allelic combination studied. In any event, however, the abnormal specification of the RPE domain as a future retina always lags behind the specification of the normal retina. For reasons of simplicity, we therefore use the term “transdifferentiation” not only for the single *Mitf* mutants but also for the double mutants.

Despite the above mentioned exacerbation of the RPE-to-retina specification, dorso-ventral polarity was maintained because neither did a dorsal marker, *Tbx5*, extend ventrally nor a ventral marker, *Vax2*, extend dorsally in E13.5 *Pax6^Sey-Neu^/Pax6^+^*; *Mitf^mi-vga9^/Mitf^mi-vga9^* double mutants (Figure S6G–S6J). That the ventral RPE was unaffected in the *Pax6/Mitf* double mutants (Figure S6I, S6J) was likely due to the fact that *Vax1/2* and *Nr2f1/2*, two genes whose alterations lead to ventral and central RPE transdifferentiation [26], [27], were wild type in these mice. Furthermore, at P0, the transdifferentiating RPE domain of *Pax6^Sey-Neu^*/*Pax6* ^+^; *Mitf^mi-ΔD^/Mitf^mi-ΔD^* (Figure 4) and *Pax6^Sey-Neu^*/*Pax6* ^+^; *Mitf^mi-vga9^/Mitf^mi-vga9^* mutants (Figure S7) eventually generated differentiated retinal cells, including ISL1-positive ganglion cells, NF160-positive horizontal cells, and VC1.1-positive amacrine cells. They assumed a laminar structure similar to that seen in the normal retina, except that, as expected based on the developmental topology, the lamination was inverted relative to that of the retina [7]. In contrast, in *Mitf* mutants carrying the *Pax6* YAC transgene, the RPE maintained its normal morphology as a monolayer. Taken together, the above results indicated that in two distinct *Mitf* mutants, a reduction in *Pax6* gene dose promotes formation of a retina from the neuroepithelial domain that normally becomes RPE while an increase in *Pax6* gene dose interferes with such a transition.

### *Mitf* and *Pax6* Regulate the Expression of the *Mitf* Paralog *Tfec*

**Figure 5:**
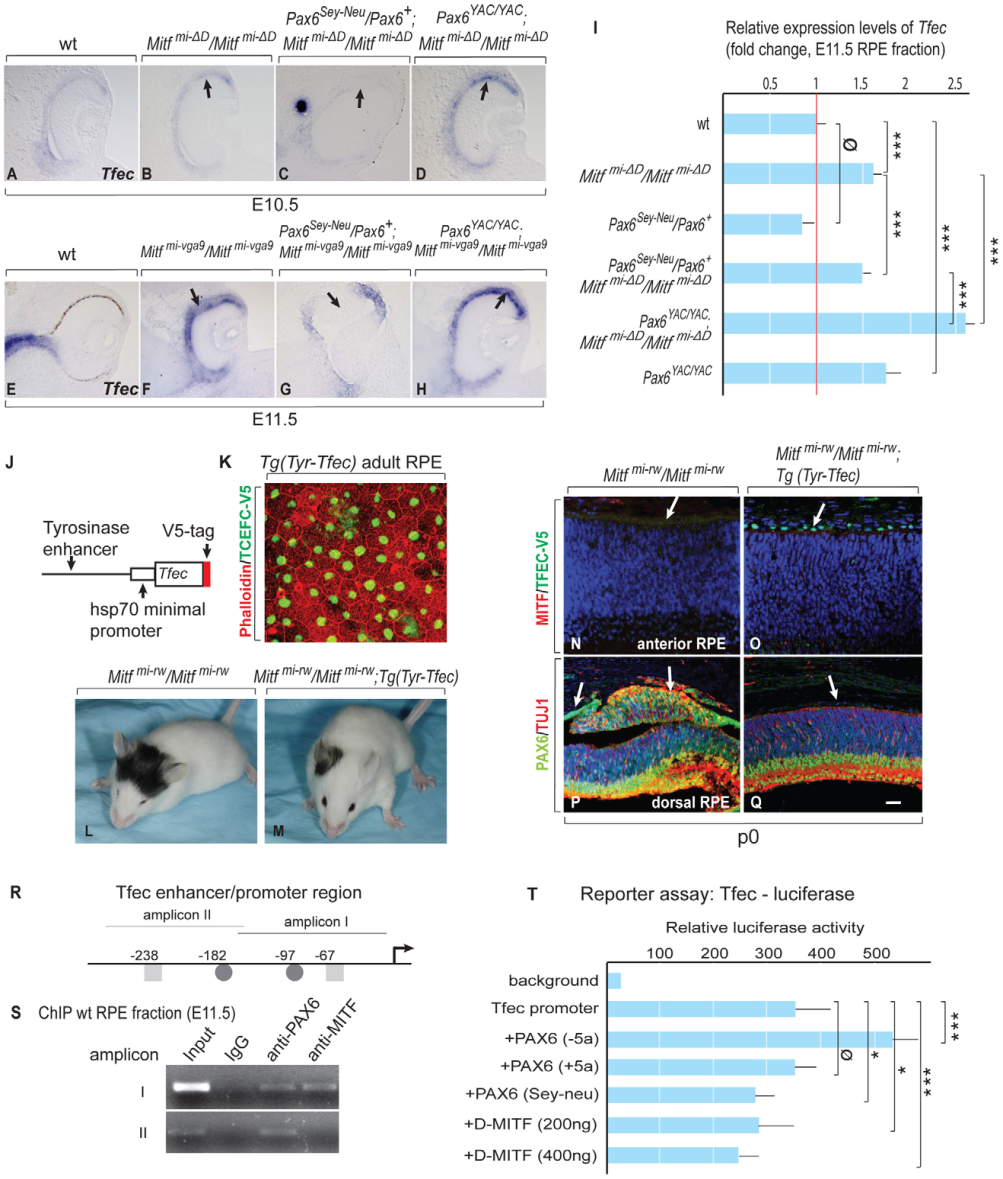
*Tfec* is regulated by *Pax6* and compensates an *Mitf* mutation in the RPE. (A–D) In situ hybridization for *Tfec* in embryonic eyes of the indicated genotypes and developmental time points. Arrows mark areas with altered *Tfec* expression compared to wild type. (I) Quantitative RT-PCR analysis of *Tfec* mRNA levels in E11.5 RPE fractions obtained from the indicated mutants. Means and S.D. based on 3 biologically independent samples. Statistical significance shown as for Figure 3. (J) Schematic diagram of the Tyrosinase enhancer-hsp70 minimal promoter-Tfec-V5 expression cassette used for generating transgenic mice. (K) Flat mount of adult RPE from *Tyr-Tfec* transgenic line stained with Phalloidin (green) and anti-V5 antibody (red). (L,M) Transgenic TFEC rescues eye defects in *Mitf^mi-rw^/Mitf^mi-rw^* mice. The eyes of *Tg (Tyr-Tfec);Mitf^mi-rw^/Mitf^mi-rw^* mice (n = 18) are bigger than those of *Mitf^mi-rw^/Mitf^mi-rw^* mice (n = 16). (N–Q) Transgenic TFEC expression suppresses RPE-retina transdifferentiation in *Mitf^mi-rw^/Mitf^mi-rw^* mice. P0 mouse eye sections stained as shown, with nuclei stained with Topo3. (N,O) TFEC-V5 staining is seen only in *Tg (Tyr-Tfec); Mitf^mi-rw^/Mitf^mi-rw^* mice (arrows) and MITF staining is below threshold in this area of the RPE. (P,Q) Note absence of ectopic PAX6 and TUJ1 expression in the RPE of *Tg (Tyr-Tfec); Mitf^mi-rw^/Mitf^mi-rw^* mice. Scale bar (A–H): 110 µm; (N–Q): 90 µm. (R) Schematic diagram of the *Tfec* enhancer/promoter region. The positions of conserved potential binding sites for MITF (▪) and PAX6 (•) are given relative to the translation start site of *Tfec* isoform A [30]. (S) ChIP assays of wild-type RPE fractions dissected from E11.5 embryonic eyes, using PAX6 and MITF-specific antibodies. (T) A 700 bp *Tfec* enhancer/promoter region containing amplicons I and II (see R) was used for reporter assays in ARPE19 cells co-transfected with expression plasmids for the indicated transcription factors. Results represent normalized mean luciferase activity units obtained from 9 independent transfections. S.D. and statistical significance are indicated.

It has previously been shown that the *Mitf* paralog *Tfec* is expressed in the developing RPE and that its expression is increased by homozygosity for *Mitf^mi-vga9^* [28]. *Tfec* shares with *Mitf* a similar multi-promoter gene structure and encodes proteins with a basic-helix-loop-helix domain that is nearly identical with that of MITF [4], suggesting the two proteins can form heterodimers and share an overlapping set of target genes. We confirmed the increased expression of *Tfec* in the RPE of E11.5 *Mitf^mi-vga9^* homozygotes by in situ hybridization (Figure 5E, 5F) and determined by qRT-PCR that this increase was 2.5-fold (data not shown). *Tfec* was, therefore, an excellent candidate gene that might participate in the above interplay between *Mitf* and *Pax6* mutations. In fact, by in situ hybridization, *Tfec* was weakly upregulated in the RPE of E10.5 homozygous *D-Mitf* knock-outs (Figure 5A, 5B), downregulated when they were heterozygous for *Pax6^Sey-Neu^* (Figure 5C), and upregulated when they were transgenic for *PAX6* YAC (Figure 5D). Quantitative RT-PCR confirmed that *Tfec* was upregulated about 1.7-fold in the E11.5 RPE of homozygous *D-Mitf* knock-outs, slightly, but significantly less when they carried the *Pax6^Sey-Neu^* allele, and significantly more when they carried the *PAX6* YAC transgene (Figure 5I). Similar observations were made for *Mitf^mi-vga9^/Pax6-*mutant combinations by in situ hybridization even though the *PAX6* YAC-mediated upregulation was not as striking as that in *D-Mitf* knock-outs (Figure 5E–5H). These results suggest that MITF regulates *Tfec* negatively in the RPE while PAX6 regulates it positively.

To directly demonstrate that TFEC can rescue eye defects in an *Mitf* mutant, we generated lines of transgenic mice that express a V5-tagged *Tfec* cDNA under control of an RPE-specific enhancer of the *Tyrosinase* gene [29], and crossed them with *microphthalmia red-eyed white* (*Mitf^mi-rw^*) mice (Figure 5J–5Q). We chose this hypomorphic *Mitf* allele (rather than *Mitf^mi-vga9^*) because *Mitf^mi-rw^* homozygotes express small amounts of *Tyrosinase* and functional, though aberrant MITF protein, likely enough to stimulate the transgenic *Tyrosinase* enhancer, and yet they have an obvious small-eye phenotype [14]. Indeed, the *Tyr-Tfec* transgene was able to rescue eye size (Figure 5L, 5M) and RPE abnormalities (Figure 5N–5Q) of *Mitf^mi-rw^* homozygotes. Importantly, this rescue was not due to upregulation of the endogenous MITF (Figure 5O). Only minimal rescue in eye size was observed with transgenic lines expressing TFEC at lower levels (not shown). Hence, transgenic TFEC can replace MITF in the RPE at least with respect to its anti-proliferative functions.

To analyze whether PAX6 and MITF regulate *Tfec* directly, we employed chromatin immunoprecipitation (ChIP) and reporter assays, using a 5 kbp *Tfec* enhancer/promoter sequence. Based on expression analysis of *Tfec* RNA isoforms [30], this sequence corresponds to the upstream region of the only isoform we found expressed in RPE. It contains a number of conserved potential PAX6 and MITF binding sites that we arbitrarily grouped into six amplicons. ChIP analyses of wild-type RPE fractions showed PAX6 and MITF binding to amplicon I, PAX6-only binding to amplicon II (Figure 5R, 5S), and binding of neither protein to the other four amplicons (not shown). In reporter assays in the human RPE cell line ARPE19, a 700 bp promoter element containing amplicons I and II was spontaneously active. To test whether this reporter fragment could be further stimulated by PAX6, we expressed either one of two alternatively spliced *Pax6* isoforms, *Pax6 (+5a)* and *Pax6 (−5a*), which differ by a 14-amino acids insertion in the paired domain and exhibit unique DNA binding properties [22], [23]. In fact, the *Tfec* promoter was stimulated by a vector expressing the PAX6 (−5a) but not the PAX6 (+5a) isoform. Transfection of a mutant *Pax6* (−5a) cDNA that represents the *Pax6^Sey-Neu^* allele (stop codon at amino acid position 301) (18) did not stimulate the *Tfec* promoter. Importantly, transfection of a *D-Mitf* expression vector reduced the activity of the *Tfec* promoter in a dose-dependent manner, suggesting that *Mitf* can indeed repress *Tfec* (Figure 5T). These results suggest that in vivo, *Tfec* is regulated at least in part directly by PAX6/MITF and participates in the circuit that can partially compensate for the reduction or loss of MITF function, thus supporting *Pax6*'s anti-retinogenic activities in the RPE.

### Gene Expression Profiles in the Transdifferentiating RPE

To gain deeper insights into the underlying mechanisms of PAX6/MITF/TFEC-mediated RPE transdifferentiation, we performed a microarray analysis on the Affymetrix platform (Affymetrix Mouse Gene 1.0 ST). For this analysis, we used cDNAs prepared from E11.5 RPE fractions from the indicated *D-Mitf* knock-outs differing in their *Pax6* gene dose, and from their respective controls. We centered the evaluation of up- and downregulated genes on *Pax6^Sey-Neu^/Pax6^+^; Mitf^mi-ΔD^/Mitf^mi-ΔD^* RPE fractions because their RPEs display the greatest extent of transdifferentiation. Table 1 and Table 2 show a selection of genes upregulated at least 1.49-fold in such RPE fractions and downregulated at least 1.43-fold when compared to wild-type RPE fractions (for full data and technical details, see Table S2). The microarray assay confirmed upregulation of retinal genes, including *Vsx2*, *Rax*, *Pax6* and *Six6*, and downregulation of RPE genes, including *Tyrp1*, *Silv*, *Tyr* and *Mitf*, and showed the expected changes of these genes under the additional genetic configurations. Importantly, among the many genes whose expression changes in *Pax6^Sey-Neu^/Pax6^+^*; *Mitf^mi-ΔD^/Mitf^mi-ΔD^* RPEs, we found prominent upregulation of two genes that encode extracellular ligands and that are potentially involved in eye development. One, *Fgf15*, is a fibroblast growth factor whose paralogs have previously been shown to promote retinal at the expense of RPE development [31], [32]. The other, *Dkk3*, is a member of a family of genes involved in the inhibition of WNT signaling [33], which is known to promote RPE development [8], [11]. *Dkk3*'s role in WNT signaling is not entirely clear, however, as in several cancer cell lines, WNT signaling is increased after *Dkk3* downregulation [34] while in a Müller glia cell line (though not in cos7 cells), it is increased after *Dkk3* upregulation [35]. This suggests that *Dkk3* acts in a context-dependent way, prompting us to focus specifically on FGF15 and DKK3 in RPE transdifferentiation.

**Table 1:** Gene expression profiling in mutant E11.5 RPE fractions.

Selected genes upregulated in *Pax6^Sey-Neu^/Pax6^+^; Mitf^ mi-ΔD^/Mitf ^mi-ΔD^* as compared to wild type (fold change)							
Gene name	Gene Symbol	Gene ID	*Pax6^Sey-Neu^/Pax6* ^+^	*Mitf^mi-^* ^Δ*D*^ */Mitf^mi-^* ^Δ*D*^	*Pax6^Sey-Neu^/Pax6^+^; Mitf^mi-^* ^Δ*D*^ */Mitf^mi-^* ^Δ*D*^	*Pax6^YAC/YAC^; Mitf^mi-^* ^Δ*D*^ */Mitf^mi-^* ^Δ*D*^	*Pax6^YAC/YAC^*
fibroblast growth factor 15	*Fgf15*	14170	1.06	−1.04	**5.17**	1.01	1.16
visual system homeobox 2	*Vsx2*	12677	−1.41	−1.25	**3.83**	1.13	1.28
retina/anterior neural fold homeobox	*Rax*	19434	−1.04	−1.11	**3.62**	1.13	1.23
paired box 6	*Pax6*	18508	1.63	1.07	**3.03**	1.24	1.04
dickkopf homolog 3	*Dkk3*	50781	−1.15	−1.08	**2.60**	1.25	1.19
SIX homeo box6	*Six6*	20476	−1.12	1.00	**2.44**	1.57	1.38
RAR-related orphan receptor B	*Rorb*	225998	−1.24	−1.07	**2.07**	1.14	1.14
SRY-box 2	*Sox2*	20674	1.32	−1.05	**1.97**	−1.05	1.03
transcription factor EC	*Tfec*	21426	−1.15	1.87	**1.49**	2.64	1.71

**Table 2:** Gene expression profiling in mutant E11.5 RPE fractions.

Selected genes downregulated in *Pax6^Sey-Neu^/Pax6^+^; Mitf^ mi-ΔD^/Mitf ^mi-ΔD^* as compared to wild type (fold change)							
Gene name	Gene Symbol	Gene ID	*Pax6^Sey-Neu^/Pax6* ^+^	*Mitf^mi-^* ^Δ*D*^ */Mitf^mi-^* ^Δ*D*^	*Pax6^Sey-Neu^/Pax6^+^; Mitf^mi-^* ^Δ*D*^ */Mitf^mi-^* ^Δ*D*^	*Pax6^YAC/YAC^; Mitf^mi-^* ^Δ*D*^ */Mitf^mi-^* ^Δ*D*^	*Pax6^YAC/YAC^*
retinaldehyde binding protein 1	*Rlbp1*	19771	−1.00	−1.67	**−2.16**	−2.06	−1.24
tyrosinase-related protein 1	*Tyrp1*	22178	−1.04	−1.48	**−1.82**	−1.51	1.10
RAB27A	*Rab27a*	11891	−1.12	−1.40	**−1.81**	−1.09	1.28
melan-A	*Mlana*	77836	−1.11	−1.79	**−1.72**	−1.57	1.30
transthyretin	*Ttr*	22139	1.55	−2.85	**−1.72**	−2.97	−2.87
silver homolog	*Silv*	20431	−1.03	−1.31	**−1.70**	−1.25	1.06
tyrosinase	*Tyr*	22173	−1.04	−1.20	**−1.62**	−1.55	1.02
SRY-box 9	*Sox9*	20682	1.03	−1.12	**−1.60**	−1.57	−1.38
microphthalmia-associated TF	*Mitf*	17342	−1.03	−1.31	**−1.43**	−1.19	1.13

### *Fgf15* and *Dkk3* Are Coordinately Regulated by PAX6 and MITF/TFEC

**Figure 6:**
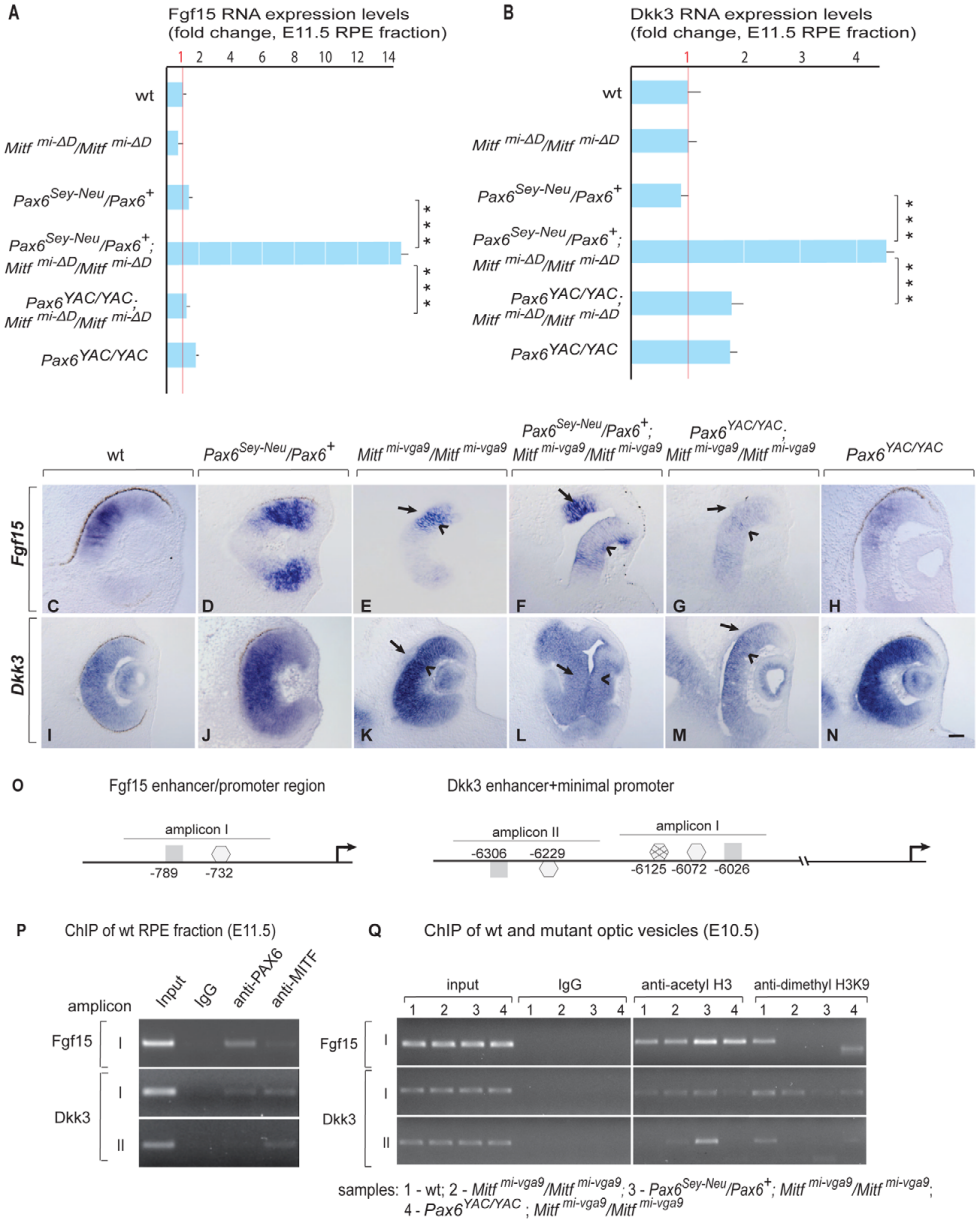
PAX6 and MITF/TFEC together suppress *Fgf15 and Dkk3* in the developing RPE. (A,B) Quantitative RT-PCR of *Fgf15* and *Dkk3* RNA of E11.5 RPE fractions of wild type and indicated mutants. Mean, S.D. and statistical significance based on 3 biological replicates. (C–N) In situ hybridization for *Fgf15* (C–H) and *Dkk3* (I–N) on eye sections of E11.5 embryos of the indicated genotypes. Arrows in E–G,K–M point to the RPE (note ectopic *Fgf15* expression in the RPE of *Pax6^Sey-Neu^/Mitf^mi-vga9^* double mutant in F and ectopic *Dkk3* expression in the RPE of *Pax6^Sey-Neu^/Mitf^mi-vga9^* double mutant in L). Scale bar (C–N):115 µm. (O) Schematic representation of *Fgf15* and *Dkk3* enhancer/promoter regions showing amplicons containing conserved potential binding sites for MITF (▪) and PAX6 (consensus 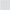, non-consensus 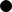), with positions indicated relative to translation start sites. (P) ChIP assays performed with indicated antibodies on RPE fractions dissected from E11.5 wild-type embryonic eyes. Amplicons as indicated in (O). (Q) ChIP assays performed with the indicated antibodies on optic vesicle tissue dissected from E10.5 embryonic eyes of the indicated genotypes. Anti-acetyl H3 signal represents active chromatin domains and anti-dimethyl H3K9 signal inactive chromatin domains. For details see text.

We first confirmed by qRT-PCR that *Fgf15* and *Dkk3* are indeed upregulated in RPE fractions of *Pax6^Sey-Neu^/Pax6^+^*; *Mitf^mi-ΔD^/Mitf^mi-ΔD^* embryos. As shown in Figure 6A, 6B, *Fgf15* expression was increased approximately 15-fold and *Dkk3* expression approximately four-fold compared to wild-type or *Mitf^mi-ΔD^/Mitf^mi-ΔD^* RPEs. Although gene expression profiling showed that another member of the *Dkk* family, *Dkk1*, was also upregulated in *Pax6^Sey-Neu^/Pax6^+^*;*Mitf^mi-ΔD^/Mitf^mi-ΔD^* RPEs (see Table S2), it did not show the prominent *Pax6* gene dose-dependent difference observed for *Dkk3* and was therefore not further analyzed. Strong expression of both *Fgf15* and *Dkk3* was also seen by in situ hybridization in the transdifferentiating RPEs of *Pax6^Sey-Neu^/Pax6^+^*;*Mitf^mi-vga9^/Mitf^mi-vga9^* embryos at E11.5, but not in the RPEs of *Pax6^YAC/YAC^*;*Mitf^mi-vga9^/Mitf^mi-vga9^* embryos (Figure 6C–6N, arrows in F,L pointing to the transdifferentiating RPE) and not at E9.5–E10 (data not shown). These results suggest that PAX6 and MITF/TFEC together normally suppress *Fgf15* and *Dkk3* in the developing RPE. Nevertheless, single reductions of either PAX6 or MITF alone have only mild effects on *Fgf15* or *Dkk3* expression, consistent with their milder phenotypes (Figure 6A–6N). The results also suggest that ectopically expressed *Fgf15* and *Dkk3* help to induce the RPE-to-retina transdifferentiation.

We next used ChIP and luciferase reporter assays similar to those shown for the *Tfec* promoter to test whether the regulation of *Fgf15* and *Dkk3* by *Pax6* and *Mitf/Tfec* is direct or indirect. Although a 5 kbp region upstream of the *Fgf15* translational start site showed various conserved potential binding sites for PAX6 and MITF, only one region, represented by amplicon I, which contains one potential PAX6 and one potential MITF binding site (Figure 6O, left side), gave ChIP signals on wild-type RPE fractions (Figure 6P and data not shown). For *Dkk3*, conserved potential binding sites for PAX6 or MITF, arbitrarily grouped into amplicons I and II (Figure 6O, right side) were present in a region between position −6026 and −6306 of the annotated start site of translation. ChIP assays with wild-type RPE fractions gave signals for both PAX6 and MITF with *Dkk3* amplicon I, but only for MITF with amplicon II (Figure 6P). Luciferase reporter assays in RPE cells (ARPE19) showed that PAX6, MITF and TFEC negatively regulate these sequence elements, though in the case of *Fgf15* only when PAX6 and MITF, or PAX6 and TFEC, were co-expressed with the reporter (Figure S8). As done for the *Tfec* promoter, we then evaluated the chromatin status of these elements in the mutants, again using whole optic vesicles. Although part of the ChIP signal in optic vesicles was likely contributed by the high level of expression of *Fgf15* and *Dkk3* in the retina, there was a substantial increase in the anti-acetyl H3 ChIP signal in *Pax6^Sey-Neu^/Pax6^+^; Mitf^mi-vga9^/Mitf^mi-vga9^* for *Fgf15* amplicon I and *Dkk3* amplicon II (lane 3 in Figure 6Q) when compared to the respective controls. Conversely, the anti-dimethyl H3K9 ChIP signal was only seen in wild-type and not in *Pax6^Sey-Neu^/Pax6^+^; Mitf^mi-vga9^/Mitf^mi-vga9^*. These results suggest that the tested promoter regions are indeed subject to in vivo regulation by PAX6 and MITF and that this regulation is at least in part direct.

### DKK3 and FGF Cooperate to Promote RPE Transdifferentiation

**Figure 7:**
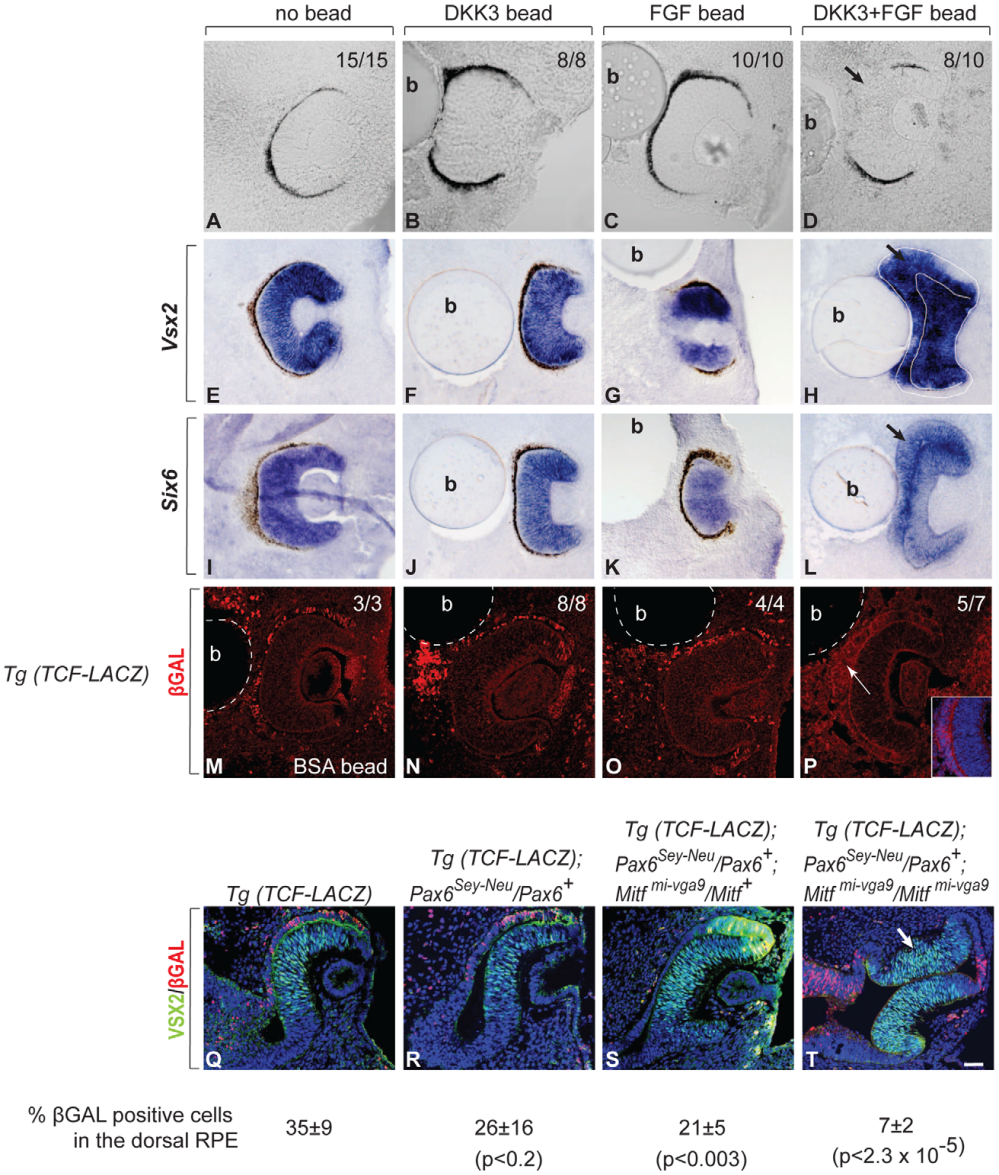
FGF and DKK3 induce RPE transdifferentiation. (A–P) Cultures of developing eyes explanted from E10.0 wild-type embryos. (A–D) RPE pigmentation develops within 48 hours in the absence of a bead (A) or in the presence of beads soaked in 0.65 µg/ml of recombinant DKK3 (B) or 0.35 µg/ml of recombinant FGF2 (C) but is segmentally missing in the vicinity of a bead soaked in a combination of 0.65 µg/ml of DKK3 and 0.35 µg/ml of FGF2 (D). The number of cultures with the represented results per total cultures tested is shown in the upper right corner. (E–L) Representative cultures were fixed, cryosectioned and subjected to in situ hybridization with the indicated probes. Note induction of the retinal factors *Vsx2* and *Six6* in the RPEs only after implantation of a DKK3/FGF2 double-coated bead (H,L). (M–P) Cultures were established from TCF-LacZ transgenic embryos and implanted with beads coated with bovine serum albumin (BSA, M) or the indicated growth factors. They were fixed, cryosectioned, and stained with antibodies to ßGAL. Note absence of ßGAL staining only in cultures implanted with double-coated beads (inset shows higher magnification of the RPE region) (P). The number of cultures with the shown results per total cultures established is shown in the right upper corner. (Q–R) VSX2/ßGAL double-labeled cryosections of E10.5 embryos of the indicated genotypes. Note absence of ßGAL labeling in the transdifferentiating portions of the RPEs in *Mitf^mi-vga9^* heterozygous or homozygous embryos when they carry a *Pax6^Sey-Neu^* allele (S,T, arrow in T). Numbers represent % ßGAL positive cells in the dorsal RPE. P values based on Student's t test. Scale bar (A–D, M–P, Q–T): 90 µm; (E–L): 115 µm.

The above results show that *Dkk3* and *Fgf15* are major targets of the circuits that regulate RPE development. To test whether DKK3 and FGF signaling are also actively involved in RPE transdifferentiation, we employed wild-type optic vesicle explant cultures into which beads soaked in human recombinant DKK3 or FGF2 (which, like FGF15 or its human ortholog FGF19, binds the same isoforms of all four main FGF receptors, [36]) were implanted. To test if FGF and DKK3 signaling can induce transdifferentiation in RPE cells, we used explant cultures established at E10.0, when the RPE fate is just specified. If left untreated or implanted with a bead coated with bovine serum albumin, these explant cultures normally develop within 48 hours into optic cups with a clearly pigmented RPE [Figure 7A, 15/15 cultures developing a pigmented RPE; see also [7]]. The implantation of beads soaked in human recombinant DKK3 at 0.65 or 1 µg/ml had little effect on RPE development (Figure 7B, 0.65 µg/ml, 8/8 cultures with pigmented RPE). Therefore, we tested for cooperation between DKK3 and FGF signaling. Previous results showed that beads soaked in 1 µg/ml of FGF2 are capable of inducing RPE transdifferentiation on their own [6], [7]. Nevertheless, a dose response curve indicated that implanting beads soaked in 0.35 µg/ml or less of FGF2 was without effect on pigmentation (Figure 7C, 0.35 µg/ml, 10/10 cultures with pigmented RPE). Beads soaked in a mixture of 0.65 µg/ml of DKK3 and 0.35 µg/ml of FGF2, however, markedly reduced pigmentation in the vicinity of the bead (Figure 7D, 8/10 cultures showing segmental RPE depigmentation). Furthermore, in situ hybridization for *Vsx2* and *Six6* showed that only the double DKK3/FGF2 treatment led to expression of these retinal genes in the RPE (Figure 7E–7L). To test whether DKK3 would inhibit the canonical WNT signaling pathway, we used optic vesicle cultures from mice transgenic for the WNT reporter Tcf-LacZ [37]. As shown in Figure 7M–7P, DKK3 beads alone could not repress ßGAL expression (nor could, for that matter, FGF2 beads alone: 8/8 and 4/4 cultures, respectively, retained ßGAL staining). However, the combination of DKK3 and FGF2 at the above concentrations efficiently repressed ßGAL expression (5/7 cultures lacking ßGAL staining).

The above results suggested that DKK3 and FGF cooperate to effect RPE transdifferentiation through the inhibition of WNT signaling and are sufficient to exert this effect. To test whether the *Pax6/Mitf* mutations (which, as shown above, upregulate *Dkk3* and *Fgf15*) also inhibit WNT signaling, we crossed the Tcf-LacZ transgene into *Pax6^Sey-Neu^/Pax6^+^* mice heterozygous or homozygous for *Mitf^mi-vga9^*. Double immunolabeling of E10.5 optic cups for VSX2 and ßGAL clearly showed that RPE transdifferentiation in *Pax6^Sey-Neu^/Pax6^+^*; *Mitf^mi-vga9^/Mitf^+^* and *Pax6^Sey-Neu^/Pax6^+^*; *Mitf^mi-vga9^/Mitf^mi-vga9^* embryos was associated with suppression of ßGAL expression, suggesting that enabling WNT signaling by inhibiting DKK3 and FGF is the common pathway through which the PAX6/MITF/TFEC regulatory circuit operates in the RPE (Figure 7Q–7T).

## Discussion

**Figure 8:**
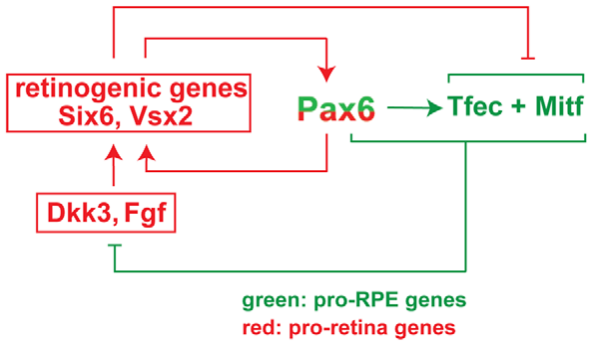
Model of the regulatory circuit involving *Pax6*, *Mitf*, and *Tcfec* during mouse RPE development.

Here we provide genetic evidence that the transcription factor PAX6, which is known in vertebrates to be crucial for the development of cornea, iris and retina, is also critical for early RPE development when tested in an *Mitf* mutant background. In fact, we find that overexpression of PAX6 in the *Mitf^mi-vga9^* null mutant background efficiently suppresses the RPE-to-retina transdifferentiation caused by Mitf-downregulation while a reduction in *Pax6* enhances this transdifferentiation. Hence, in the RPE, *Pax6* shares with *Mitf* an anti-retinogenic effect while in the retina it is pro-retinogenic. Although *Pax6* and, for that matter, many other transcription factors have evolved to play different roles in different tissues, we have to keep in mind that future retina and RPE are both derived from the same, seemingly uniform optic neuroepithelium and maintain a remarkable capacity to switch from one into the other during development and, in some vertebrates, even in adulthood [2]. As outlined below, the regulatory circuit that we here describe and schematically depict in Figure 8 may provide an explanation for the differential function of *Pax6* in retina and RPE and for the easy phenotypic switch between the two.

There is ample evidence that the initial separation of the optic neuroepithelium into future retina and RPE is effected by extracellular signals, predominantly FGFs, that emanate from the surface ectoderm. FGFs induce *Vsx2* in the distal optic vesicle domain, which in turn downregulates *Mitf* and *Tfec* and initiates the retinal developmental program [6], [28]. In contrast, BMP, ACTIVIN and WNT signaling in or around the dorso-proximal optic vesicle may stimulate *Mitf* in the dorso-proximal neuroepithelium and initiate the RPE developmental program [8], [10]–[12]. As soon as a slight bias in gene expression patterns is established to support either RPE or retina differentiation, the regulatory circuit shown in Figure 8 will reinforce this bias in the following way. Coexpression of *Pax6* with either *Mitf* or *Tfec* suppresses the expression of the retinogenic genes *Fgf15* and *Dkk3*, thus enabling canonical WNT signaling, which in turn leads to upregulation of *Mitf* and *Tfec* expression. This positive feed back loop potentiates RPE differentiation and suppresses the retinal fate. If, on the other hand, PAX6, MITF and TFEC together are unable to inhibit *Fgf15* and *Dkk3* expression, then canonical WNT signaling is inhibited and several retinogenic genes including *Six6*, *Lhx2*, and *Vsx2* are upregulated. These retinogenic genes will further stimulate the expression of *Pax6* in pro-retina and anti-RPE feed-forward loops [38], and *Mitf* expression is suppressed. Likewise, *Tfec* expression is suppressed under these conditions because the repressive effect of other retinogenic genes overcomes *Pax6's* stimulatory effect on *Tfec* (for instance, based on the analysis of *Vsx2* mutants, *Vsx2* suppresses *Tfec* in the retina despite the presence of *Pax6* [6], [28]. This regulatory circuit has the typical features of a bi-stable loop as it may assume only one of two states: either *Mitf* and/or *Tfec* are high and retinogenic genes (with the exception of *Pax6*) are low, or *Mitf* and/or *Tfec* are low, and retinogenic genes are high (Figure 8). Consequently, the neuroepithelium will develop either as a retina or as an RPE. Perturbations of the circuit, such as mutations in *Mitf* [14] or *Vsx2* [6], [28] or gene dose changes in *Pax6* could then flip the switch and lead to an inversion of cell fates rather than an indeterminate, mixed phenotype.

Experimental evidence for the above consideration is also provided by the earlier observation that physical removal of the surface ectoderm in optic vesicle cultures, and hence removal of retina-inducing signals, does not lead to the formation of two RPE-like monolayers as one might predict, but rather to a distal pigmented monolayer and a proximal retina-like tissue [7]. As this happens without deliberate addition of a source of FGFs on the proximal side, we have to assume that removal of the surface ectoderm establishes a new initial bias that is again reinforced by the *Pax6/Mitf/Tfec* regulatory circuit. It is conceivable that this is so because removal of the surface ectoderm removes not only FGF signals but other signals as well. In the chick, for instance, the dorsal portion of the surface ectoderm expresses bone morphogenic protein-4 and -7 which help initiate RPE development [10], and neural crest cells normally provide TGF-ß1 and ACTIVINS that stimulate WNT signaling in the dorsal ectoderm [9], [12]. Perhaps even more intriguing is the recent observation that optic neuroepithelial vesicles established entirely in vitro from mouse embryonic stem cells spontaneously self-organize into optic cups with properly oriented retina and RPE, without any overt presence of a typical surface ectoderm [39]. This phenomenon, too, could be explained if any initial developmental bias is reinforced by the proposed bi-stable nature of the circuit.

The above considerations, though, should not lead one to assume that the *Pax6/Mitf/Tfec* circuit is uninfluenced by other genes. A recent study showed, for instance, that in the absence of the neurogenic transcription factor SOX2, *Pax6* expression in the distal optic neuroepithelium increases, leading to conversion to ciliary epithelium but not RPE [40]. Perhaps this observation is linked to the fact that in *Mitf* single or *Pax6/Mitf* double mutants, the anterior most portion of the dorsal RPE never undergoes transdifferentiation into retina, likely because of the above mentioned RPE-inducing signals from the adjacent dorsal surface ectoderm [9], [10]. Furthermore, what we observe in mice need not necessarily apply to other vertebrates. For instance, removal of the surface ectoderm from chicken optic vesicles led to a salt and pepper structure of intermingled pigmented and non-pigmented cells, not to cleanly separated pigmented monolayers and hyperproliferating retinas as seen in mice [41]. We would predict, therefore, that even though the molecular players may be the same across all vertebrates, they may not be interconnected in the same way in all vertebrates. In fact, the PAX6 binding sites that we identified in the mouse *Tfec* promoter, although conserved across several mammalian species, are not conserved in birds and reptiles. Such differences could well explain the differences in the developmental time frame during which RPE and retina can interconvert in different species.

Finally, we would like to draw attention to the potential importance of the role of *Pax6*, *Mitf* and *Tfec* and their regulatory targets for the establishment of retinal and RPE cells from embryonic or induced pluripotent stem cells. The ability to obtain such cells in vitro has recently generated much excitement for the study of the pathogenesis of blindness caused by primary retinal or RPE malfunctions, such as adult onset macular degeneration, as well as for the eventual cell-based therapy of such diseases [42]. Although the methods to generate such cells are rapidly improving, the process is still not very well controlled, and it remains to be seen whether the in vitro generated cells represent truly authentic cell types [42]. As the production of such cells has many of the hallmarks of development, we think a careful consideration of the normal developmental pathways is paramount for their successful generation. In the light of the results presented in this paper, it would seem important, therefore, that the activity levels of PAX6 and its upstream regulators and downstream targets be carefully monitored during the in vitro generation of retinal and RPE cells.

In conclusion, we have shown that positioning of PAX6 in the center of a bi-stable regulatory loop allows this single transcription factor to be bi-functional and to participate either in a pro-retinogenic or a pro-RPE developmental pathway.

## Materials and Methods

### Mice

Extant *Pax6* and *Mitf* mutants and transgenics are described in Table S1 and were kept on a C57BL/6J background (Backcrosses: 20 for *Pax6^Sey-Neu^*, 2 for *PAX6^Yac^*, 7 for *Mitf^mi-vga9^*, strain of origin of *Mitf^mi-rw^* is C57BL/6J). C57BL/6J served as wild-type controls. *Mitf^mi-ΔD^/Mitf^mi-ΔD^* targeted mice were generated using the recombineering technology. To generate the targeting construct, the *D-Mitf* promoter/D-Mitf exon and its flanking regions (15,801 kbp) were cloned using plasmid rescue from BACRP23-9A13. A floxed neomycin resistance expression cassette flanked by 200 bp of sequence flanking the *D-Mitf* promoter/D-Mitf exon was used to replace 5.8 kbp of the *D-Mitf* promoter/D-Mitf exon from the above plasmid and used for standard targeting of LC3 ES cells (genotype [C57BL/6Nx129S6]F1), giving 6 correctly targeted colonies/40 colonies tested. Of several germline transmitting lines, one, officially designated *Mitf^tm3Arnh^*; MGI: 5050698, was selected and crossed with C57BL/6J•129S4-Prm1-Cre deleter mice (Jackson Laboratories, stock 003328, backcrossed twice to C57BL/6J). Offspring lacking the neo-cassette (*Mitf^tm3.1Arnh^*; MGI: 5050699) were backcrossed to C57BL/6J twice and then bred to homozygosity with or without the corresponding *Pax6* alleles or transgenes.

Tyr-Tfec transgenic mice (strain C57BL/6N) were generated using a construct composed of (5′-3′) a 4721 bp Tyr RPE-specific enhancer, a 985 bp hsp70 minimal promoter [29], and a 1203 bp V5-tagged Tfec cDNA. Four transgenic lines were obtained and used for crosses with *Mitf^mi-rw^* mice. Genotyping of mice was performed by Southern blot and/or PCR using primers shown in Table S3. All animal experiments were covered by approved animal protocols.

### Immunostaining and In Situ Hybridization

Immunostaining and in situ hybridizations were performed as described previously, using 16 µm thick coronal cryostat sections [14]. All eye sections are shown with the dorsal side up. Cell proliferation analysis was done by phosphohistone H3 staining of three different embryos. Immunostaining of adult mouse RPE flat-mounts was performed as described by [43]. A Zeiss LSM510 confocal (Zeiss, Thornwood, NY) and a Nikon E800 (Nikon, Melville, NY) microscope were used to record immunostainings. A Polyvar microscope (Reichert Jung, Depew, NY) was used for recording in situ hybridizations. For antibodies and in situ probes, see Tables S4 and S5.

### Expression Analysis and Whole-Genome Expression Profiling

For RNA expression, E9.5 and E10.5 optic vesicles or RPE and retinal fractions from E11.5–E18.5 embryos and P0 mice were prepared as described. RNA was extracted from pools of 20–40 individual samples and RT-PCR and real time PCR was performed according to previously published protocols [14]. For primers, see Table S1. Statistical analysis was done using two-tailed Student's t-test. Statistical significance of the data is represented as (ø) non-significant; (*) p<0.05; (**) p<0.01; and (***) p<0.001. For microarray analysis, the Affymetrix platform was used in collaboration with the NHGRI/NINDS microarray core facility and bioinformatics core.

### Chromatin Immunoprecipitation (ChIP) Assays

Pools of optic vesicles from ∼20 individual E10.5 eyes or RPE fractions from ∼30 individual E11.5 eyes were prepared as previously described [14]. ChIP assays using ChIP IT kit from Active Motif (Carlsbad, CA) was performed as described. Primers and antibodies are given in Tables S3 and S5.

### Reporter Assays

Reporter assays were performed using ARPE19 (CRL-2302, ATCC, Manassas, VA) and the dual luciferase assay kit (Promega, Madison, WI). Statistical analysis was done using two-tailed Student's t-test. Details about the plasmids used for reporter assays are given in Table S3.

### Optic Vesicle Culture

Cultures were established from E10.0 embryonic heads and maintained for 48–72 hours using published protocols [7]. Polyacrylamide beads used for implantation were soaked for an hour with BSA, recombinant DKK3 (R&D Systems, Minneapolis, MN), recombinant FGF2 (R&D Systems, Minneapolis, MN), or their combinations at the indicated concentrations. In situ hybridization/immunostaining of the vesicles was performed on 16 µm thick cryostat sections.

## Supporting Information

Figure S1 *Pax6* and *Mitf* are coexpressed during RPE development in mice. (A–H) Co-expression of PAX6 and MITF in the developing RPE. Cryostat sections of wild-type developing eyes at the indicated embryonic time points were labeled by indirect immunofluorescence using antibodies to PAX6 and MITF. (A,B) Prominent expression of both PAX6 and MITF are seen in the developing RPE at E10.5 (arrows) while only PAX6, but not MITF, is expressed in surface ectoderm, lens and retina. (C–H) PAX6 labeling in the RPE is reduced at E12.5 (C, arrow) and at E15.5 is found only in the anterior but not the central RPE segment (E,G, arrows). In contrast, MITF labeling in the RPE is retained through E15.5 in both central and anterior segment (D,F,H, arrows) and only after birth is gradually reduced, beginning in the central domain (not shown). Scale bar (C,D): 115 µm; (A,B) 90 µm; (E–H) 40 µm.
(TIF)

Figure S2 *Pax6* and *Mitf* suppress neurogenesis in the E11.5 RPE in a gene dose-dependent manner. (A,B) Immunofluoresence staining for PAX6 (green) and CD138 (red) shows increased PAX6 staining and a mild CD138 upregulation in *Mitf^mi-vga9^/Mitf^mi-vga9^* RPE (arrow in B). (C,D) Reduced *Pax6* gene dose in the *Mitf* mutant background results in more severe RPE transdifferentiation. PAX6/CD138 double-labeled eye sections from *Pax6^Sey-Neu^* heterozygous embryos with either wild-type *Mitf* (*Pax6^Sey-Neu^/Pax6^+^;* C) or mutant *Mitf* (*Pax6^Sey-Neu^/Pax6^+^; Mitf^mi-vga9^/Mitf^mi-vga9^*; D). (E,F) Similar labeling of sections from embryos with increased *Pax6* gene dose and wild-type *Mitf* (*Pax6^YAC/YAC^*; E) or mutant *Mitf* (*Pax^YAC/YAC^; Mitf^mi-vga9^/Mitf^mi-vga9^*; F). (G–J) Cell proliferation in the RPE of *Mitf^mi-vga9^* homozygotes changes with changing *Pax6* gene dose. Representative eye sections from wild type (G), *Mitf^mi-vga9^/Mitf^mi-vga9^* (H), *Pax6^Sey-Neu^/Pax6^+^; Mitf^mi-vga9^/Mitf^mi-vga9^* (I), and *Pax6^YAC/YAC^; Mitf^mi-vga9^/Mitf^mi-vga9^* (J) mutants stained with anti-phosphohistone H3 (PH3) antibody (green). Scale bar (A–F) 90 µm; (G–J): 115 µm. (K) Quantification of PH3 labeling, including results from *Pax6^Sey-Neu^/Pax6^+^* and *Pax6^YAC/YAC^* single mutants. Each bar represents the mean percentage of PH3 positive cells/total cells counted in RPE sections obtained from three different embryos. Error bars represent S.D. Statistical significance of pairwise comparisons is indicated (see Experimental Procedures).
(TIF)

Figure S3 Microdissection allows for separation of optic vesicles and eye cups into RPE and retinal fractions. Optic vesicles (OV) or eye cups were microdissected as previously described (Bharti et al., 2008). RNA was then prepared from wild-type optic vesicles (E9.5–E10), RPE + mesenchyme/choroid (E11.5-P0), and retina (E11.5-P0) and subjected to RT-PCR (Bharti et al., 2008) for quality control of tissue separation. Expression analysis of eye progenitor transcription factors (*Six3*, *Six6*, *Rax*, *Vsx2*) and RPE-specific *cadherin (P-cadherin)* was performed and β-actin was used for control purposes. As expected from previous expression data (Martinez-Morales et al., 2004), *Otx2* and *Pax6* were present in both fractions (Figure S2; note that *Pax6* gave two bands corresponding to the exon 5a+ and exon 5a- splice isoforms whose relative distribution changed in both RPE and retina between E19.5 and P0, as anticipated from previous studies (Singh et al., 2002). *Six3*, *Six6*, and *Rax* are expressed in the optic vesicle and predominantly in the retinal fractions; *P-cadherin* is predominantly expressed in the RPE fractions; and *Vsx2* exclusively in the retinal fractions.
(TIF)

Figure S4 Ectopic expression of retinal progenitor transcription factors in the RPE is regulated by *Pax6* and *Mitf* gene dose. (A–F′) Eye sections from E11.5 embryos of the indicated genotypes were subjected to in situ hybridization with the indicated probes. (G′–L′) immunofluorescent labeling of eye sections from E11.5 embryos of the indicated genotypes with SOX2 antibodies. Arrows mark the regions of the RPE that transdifferentiate in *Mitf/Pax6* double mutants or remain normal in *Mitf* mutants homozygous for the YAC transgene. Scale bar: 110 µm. (M′) RPE fractions of E11.5 embryos of the indicated genotypes were subjected to quantitative RT-PCR analysis of *Vsx2*, *Rax*, and *Six6*. All values are normalized using *Usf1*. Mean values, S.D. and statistical significance based on 3 biologically independent samples (each representing approximately 40 RPE fractions). Results are shown as fold change in RNA expression levels compared to the corresponding values from wild-type. Note that reduction in *Pax6* gene dose in *Pax6^Sey-Neu^/Pax6^+^;Mitf^mi-ΔD^/Mitf^mi-ΔD^* mutants results in a 4–8 fold upregulation of retinal progenitor factors, whereas an increase in *Pax6* gene dose in *Pax6^YAC/YAC^;Mitf^mi-ΔD^/Mitf^mi-ΔD^* mutants suppresses this upregulation.
(TIF)

Figure S5 Ectopic expression of retinal progenitor transcription factors at the optic vesicle stage. (A–F) Eye sections from E10.0–E10.25 embryos of the indicated genotypes were subjected to in situ hybridization with the indicated probes. Arrows mark the RPE. Scale bar: 60 µm.
(TIF)

Figure S6 Only the dorsal RPE of E13.5 *Pax6^Sey-Neu^/Pax6^+^;Mitf^mi-vga9^/Mitf^mi-vga9^* mutants shows transdifferentiation towards a second retina. (A,B) Expression of *connexin 43*, an RPE-marker, is affected only in the dorsal RPE of *Pax6^Sey-Neu^/Pax6^+^;Mitf^mi-vga9^/Mitf^mi-vga9^* mutants. Arrow in (B) marks dorsal transdifferentiating portion, and open arrowhead ventral, non-transdifferentiating portion. (C–F) The retinal progenitor transcription factors *Six6* and *Sox2* are expressed in transdifferentiating dorsal RPE. In situ hybridization for *Six6* or immunofluorescence for SOX2 show expression in the normal retina (solid arrowhead in D,F) and in transdifferentiated dorsal RPE (arrow in D,F). While *Six6* expression can also be seen in non-transdifferentiated ventral RPE of double mutants (open arrowhead, D), SOX2 expression is absent from this region (open arrowhead, F). (G–J) Transdifferentiated RPE maintains its dorso-ventral polarity. In situ hybridization for *Tbx5*, a dorsal retina marker, and *Vax2*, a ventral retina marker, performed on E13.5 eye sections from *Pax6/Mitf* double mutants (arrows in H,J). Scale bar (A–D, G–J):115 µm; (E–F): 90 µm.
(TIF)

Figure S7 Development of a differentiated laminated retina in *Pax6^Sey-Neu^/Pax6^+^;Mitf^mi-vga9^/Mitf ^mi-vga9^* but not *Pax6^YAC/YAC^;Mitf ^mi-vga9^/Mitf ^mi-vga9^* mice. Sections of eyes from P0 mice of the indicated genotypes were subjected to in situ hybridization for *Crx*, a photoreceptor marker (A–C) or *Math3*, an amacrine cell marker (D–F). Note that the RPE of *Mitf^mi-vga9^/Mitf^mi-vga9^* mutants weakly expresses these two markers (see higher magnification of inset images) and ectopic staining is not present in the RPE of *Pax6^YAC/YAC^;Mitf^mi-vga9^/Mitf^mi-vga9^* mutants (compare arrows in A,B and D,E with C,F for ectopic staining; arrowheads mark normal retinal staining). (G–R) Immunofluorescent labeling for the indicated markers on P0 eye sections of the indicated genotypes. ISL1 is a ganglion cell marker (G–I), as is PAX6 at this time point (J–L, P–R, green). NF160 marks horizontal cells (J–L, red); VC1.1 marks amacrine cells (M–O, red); and SYNTAXIN marks synapses (P–R, red). Arrows mark the transdifferentiating portions of the RPE in *Pax6^Sey-Neu^/Pax6^+^;Mitf^mi-vga9^/Mitf^mi-vga9^* mice (H,K,N,Q) or the corresponding non-transdifferentiating portions in *Pax6^YAC/YAC^;Mitf ^mi-vga9^/Mitf^mi-vga9^* mice (I,L,O,R). The normal retinas continue to express each of these markers (arrowheads in the corresponding figures). Scale bar (A–F): 115 µm; (G–R): 90 µm.
(TIF)

Figure S8 PAX6, MITF and TFEC regulate the activity of *Fgf15* and *Dkk3* promoter/enhancer regions in luciferase reporter assays. (A) A 1450 bp *Fgf15* enhancer/promoter region was cloned in a vector containing the luciferase reporter and transfected into ARPE19 cells along with the indicated expression vectors. Each bar represents the mean luciferase activity units obtained from 8 independent transfections after normalization with a co-transfected control renilla luciferase construct. Error bars indicate S.D. and statistical significance is given for pairwise comparisons relative to promoter-only sample. (B) A 572 bp *Dkk3* distal enhancer region was cloned upstream of 665 bp of the minimal *Dkk3* promoter region and used as in (A).
(TIF)

Table S1 *Pax6* and *Mitf* alleles used in this study. Schematics of *Pax6* and *Mitf* genomic loci, alleles used in this study, and a brief description of alleles is provided.
(DOC)

Table S2 Affymetrix microarray and bioinformatics analysis. RNA was prepared from RPE-fractions from three biological replicates each for wild type and the different *Pax6/Mitf* mutants and checked for integrity using bioanalyzer. It was then used for hybridization with Affymetrix Mouse Gene 1.0 ST Chip. Raw data were processed using Robust Multi-Array and analyzed using Genespring 7.0 software (Agilent, Santa Clara, CA). Data were normalized to the statistical mean of all detectable probe sets and its statistical significance was tested by ANOVA analysis. A total of 532 gene fragments were selected based on a maximum coefficient of variation of 1.5 and turkey p-values less than 0.05.
(DOCX)

Table S3 List of used primers. List of primers used to generate *Mitf^mi-ΔD^/Mitf^mi-ΔD^* and *Tfec* overexpressing transgenic mice, to perform genotyping, RT-PCR, and ChIP assays, and to construct reporter plasmids is listed.
(DOCX)

Table S4 List of used in situ hybridization probes. In situ probes used in this study are listed.
(DOCX)

Table S5 List of used antibodies. Antibodies used in this study, their commercial source, species and dilution is listed.
(DOCX)
