# Supplementary figures and images for: A Regulatory Loop Involving PAX6, MITF, and WNT Signaling Controls Retinal Pigment Epithelium Development
Source: PLoS Genet. 2012 Jul 5;8(7):e1002757. doi: 10.1371/journal.pgen.1002757 (PMC3390378; doi:10.1371/journal.pgen.1002757)

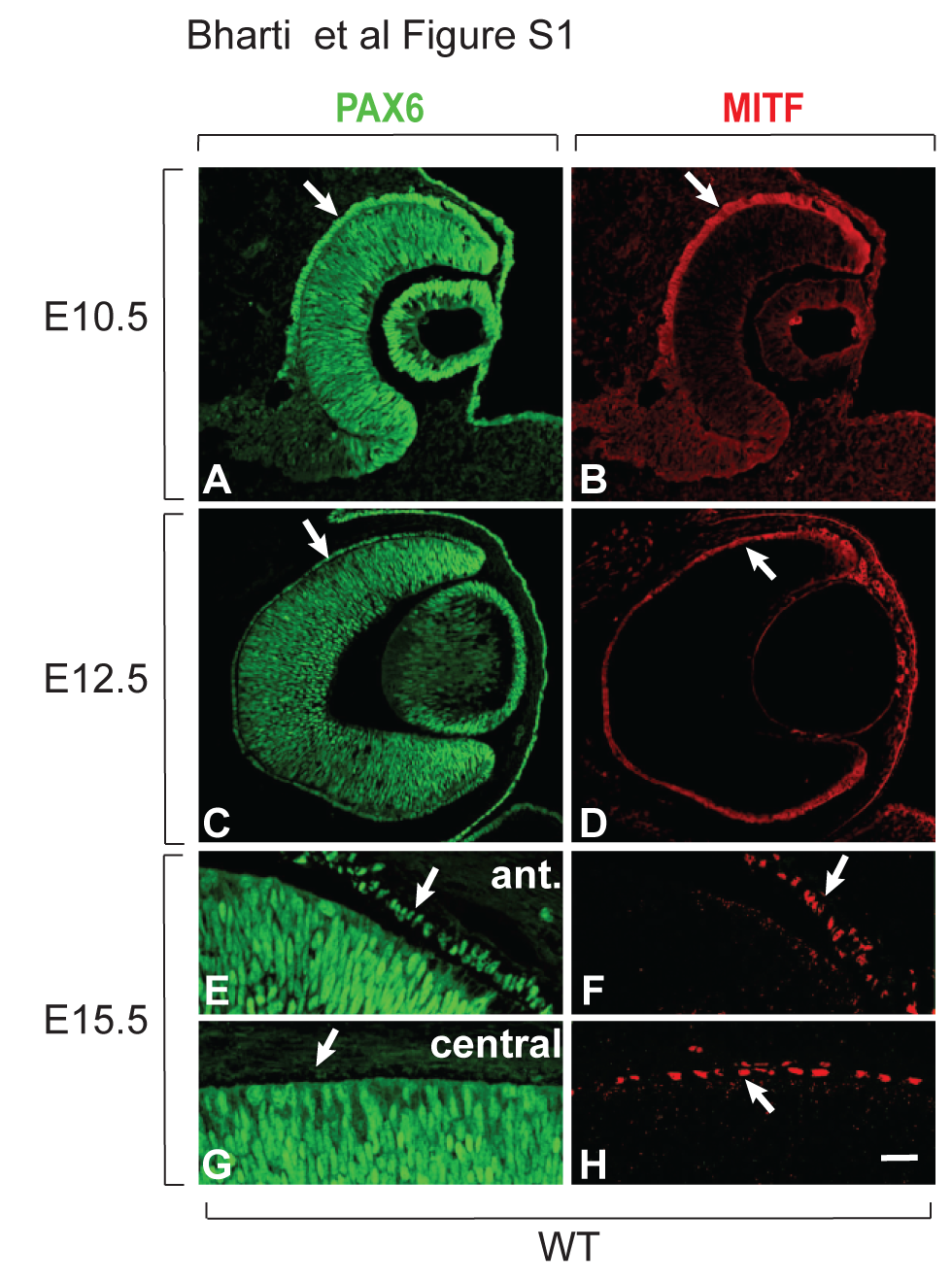

Supplement: Figure S1 — Pax6 and Mitf are coexpressed during RPE development in mice. (A–H) Co-expression of PAX6 and MITF in the developing RPE. Cryostat sections of wild-type developing eyes at the indicated embryonic time points were labeled by indirect immunofluorescence using antibodies to PAX6 and MITF. (A,B) Prominent expression of both PAX6 and MITF are seen in the developing RPE at E10.5 (arrows) while only PAX6, but not MITF, is expressed in surface ectoderm, lens and retina. (C–H) PAX6 labeling in the RPE is reduced at E12.5 (C, arrow) and at E15.5 is found only in the anterior but not the central RPE segment (E,G, arrows). In contrast, MITF labeling in the RPE is retained through E15.5 in both central and anterior segment (D,F,H, arrows) and only after birth is gradually reduced, beginning in the central domain (not shown). Scale bar (C,D): 115 µm; (A,B) 90 µm; (E–H) 40 µm. (TIF) [file pgen.1002757.s001.tif]

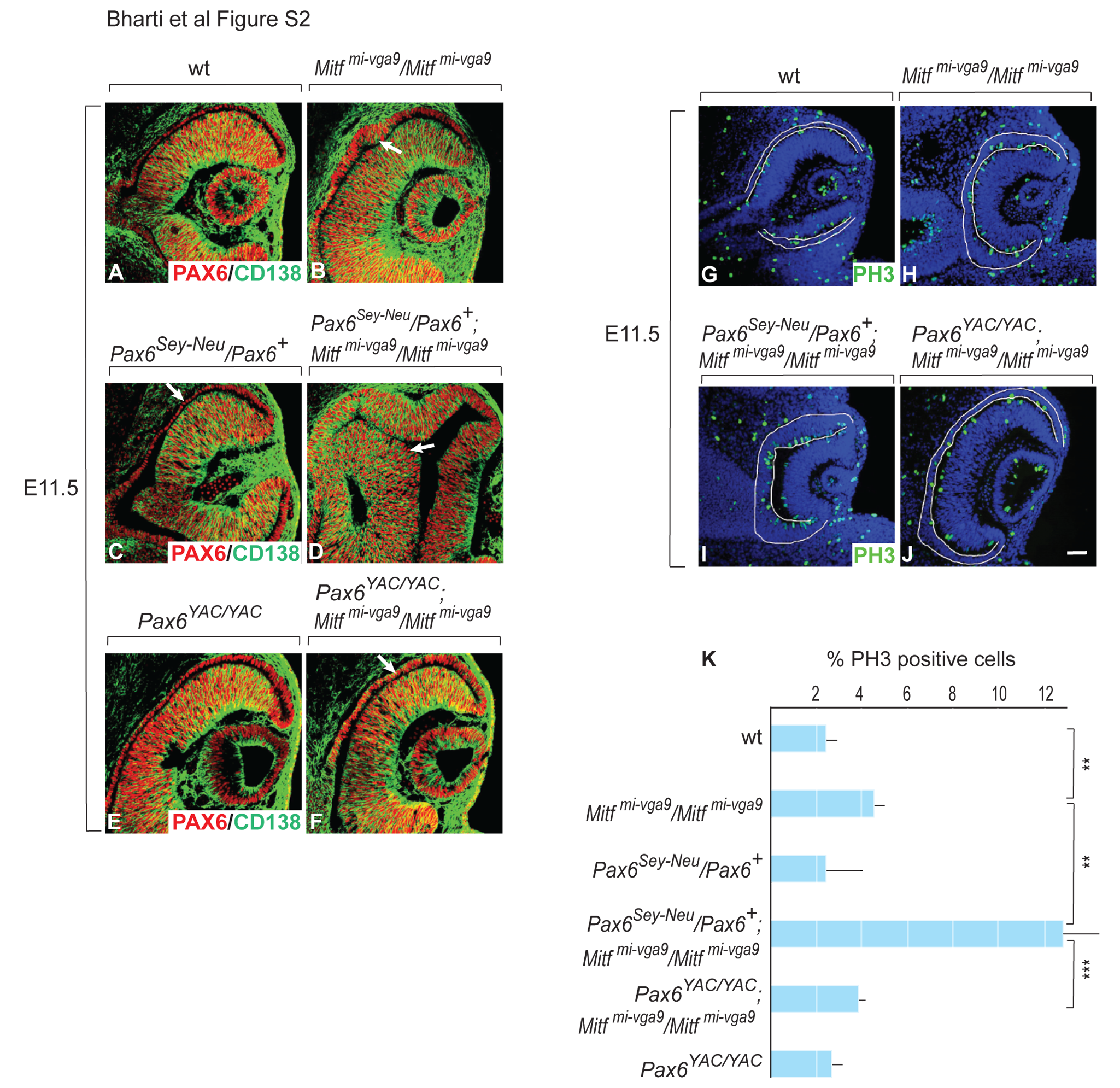

Supplement: Figure S2 — Pax6 and Mitf suppress neurogenesis in the E11.5 RPE in a gene dose-dependent manner. (A,B) Immunofluoresence staining for PAX6 (green) and CD138 (red) shows increased PAX6 staining and a mild CD138 upregulation in Mitfmi-vga9/Mitfmi-vga9 RPE (arrow in B). (C,D) Reduced Pax6 gene dose in the Mitf mutant background results in more severe RPE transdifferentiation. PAX6/CD138 double-labeled eye sections from Pax6Sey-Neu heterozygous embryos with either wild-type Mitf (Pax6Sey-Neu/Pax6+; C) or mutant Mitf (Pax6Sey-Neu/Pax6+; Mitfmi-vga9/Mitfmi-vga9; D). (E,F) Similar labeling of sections from embryos with increased Pax6 gene dose and wild-type Mitf (Pax6YAC/YAC; E) or mutant Mitf (PaxYAC/YAC; Mitfmi-vga9/Mitfmi-vga9; F). (G–J) Cell proliferation in the RPE of Mitfmi-vga9 homozygotes changes with changing Pax6 gene dose. Representative eye sections from wild type (G), Mitfmi-vga9/Mitfmi-vga9 (H), Pax6Sey-Neu/Pax6+; Mitfmi-vga9/Mitfmi-vga9 (I), and Pax6YAC/YAC; Mitfmi-vga9/Mitfmi-vga9 (J) mutants stained with anti-phosphohistone H3 (PH3) antibody (green). Scale bar (A–F) 90 µm; (G–J): 115 µm. (K) Quantification of PH3 labeling, including results from Pax6Sey-Neu/Pax6+ and Pax6YAC/YAC single mutants. Each bar represents the mean percentage of PH3 positive cells/total cells counted in RPE sections obtained from three different embryos. Error bars represent S.D. Statistical significance of pairwise comparisons is indicated (see Experimental Procedures). (TIF) [file pgen.1002757.s002.tif]

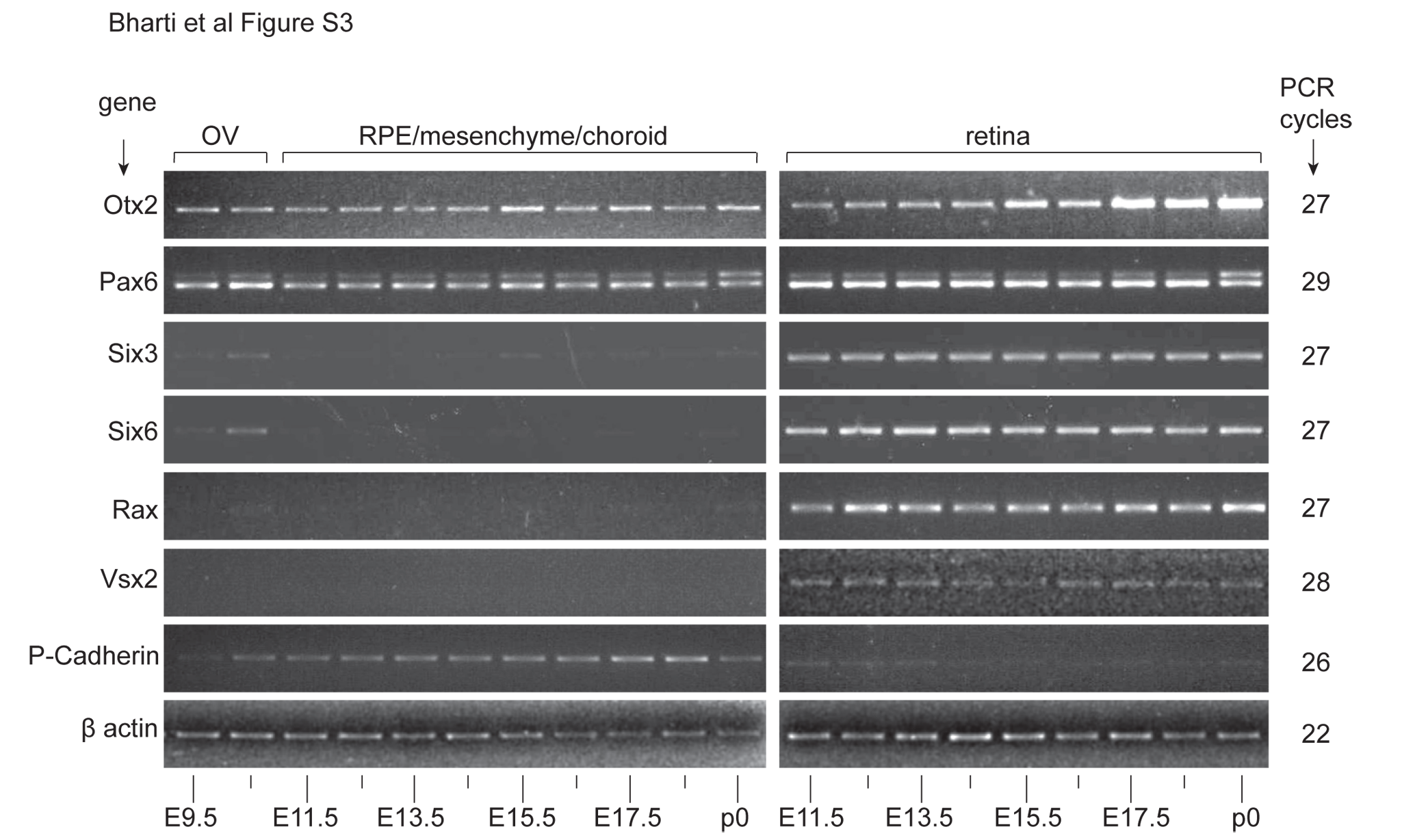

Supplement: Figure S3 — Microdissection allows for separation of optic vesicles and eye cups into RPE and retinal fractions. Optic vesicles (OV) or eye cups were microdissected as previously described (Bharti et al., 2008). RNA was then prepared from wild-type optic vesicles (E9.5–E10), RPE + mesenchyme/choroid (E11.5-P0), and retina (E11.5-P0) and subjected to RT-PCR (Bharti et al., 2008) for quality control of tissue separation. Expression analysis of eye progenitor transcription factors (Six3, Six6, Rax, Vsx2) and RPE-specific cadherin (P-cadherin) was performed and β-actin was used for control purposes. As expected from previous expression data (Martinez-Morales et al., 2004), Otx2 and Pax6 were present in both fractions (Figure S2; note that Pax6 gave two bands corresponding to the exon 5a+ and exon 5a- splice isoforms whose relative distribution changed in both RPE and retina between E19.5 and P0, as anticipated from previous studies (Singh et al., 2002). Six3, Six6, and Rax are expressed in the optic vesicle and predominantly in the retinal fractions; P-cadherin is predominantly expressed in the RPE fractions; and Vsx2 exclusively in the retinal fractions. (TIF) [file pgen.1002757.s003.tif]

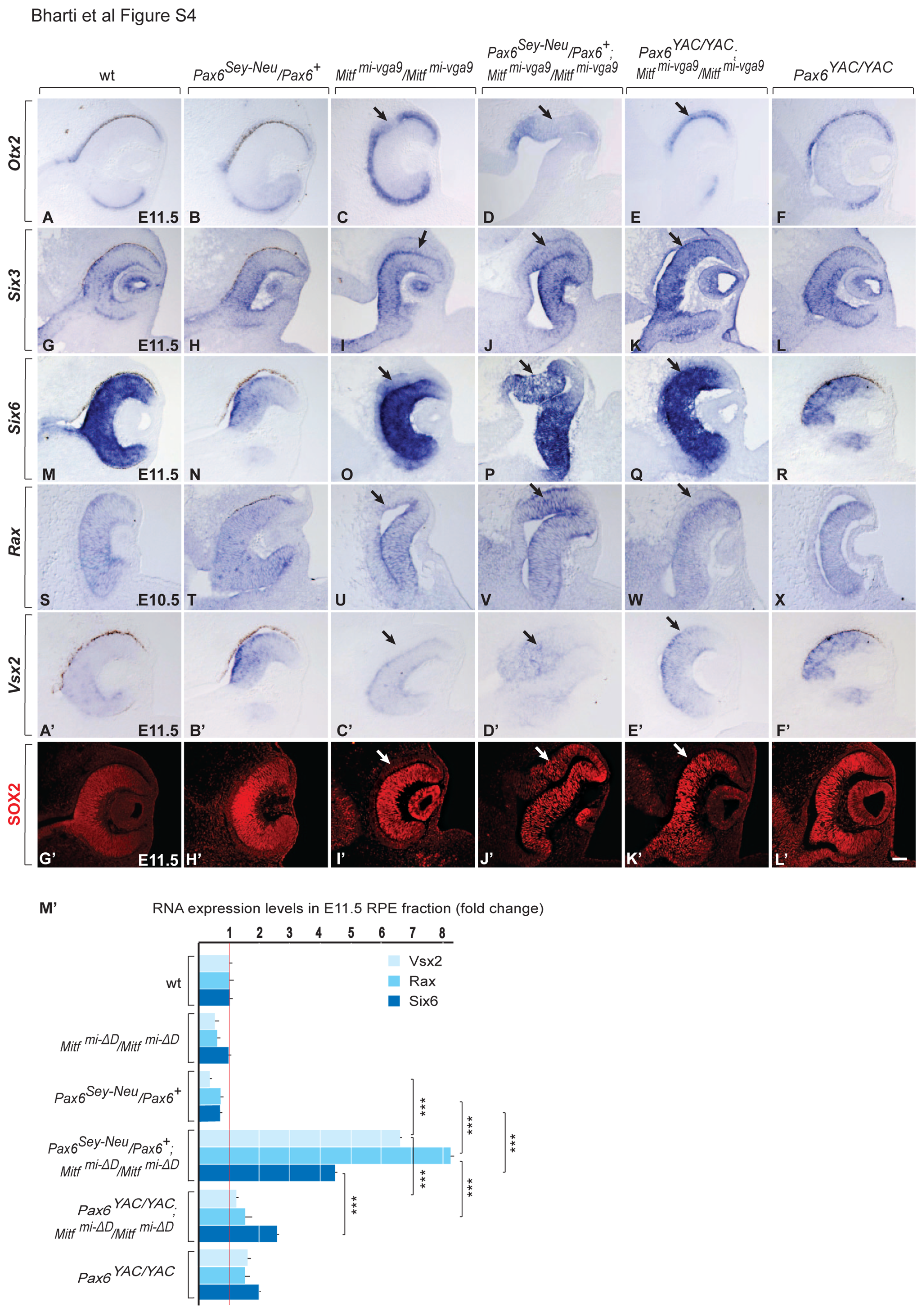

Supplement: Figure S4 — Ectopic expression of retinal progenitor transcription factors in the RPE is regulated by Pax6 and Mitf gene dose. (A–F′) Eye sections from E11.5 embryos of the indicated genotypes were subjected to in situ hybridization with the indicated probes. (G′–L′) immunofluorescent labeling of eye sections from E11.5 embryos of the indicated genotypes with SOX2 antibodies. Arrows mark the regions of the RPE that transdifferentiate in Mitf/Pax6 double mutants or remain normal in Mitf mutants homozygous for the YAC transgene. Scale bar: 110 µm. (M′) RPE fractions of E11.5 embryos of the indicated genotypes were subjected to quantitative RT-PCR analysis of Vsx2, Rax, and Six6. All values are normalized using Usf1. Mean values, S.D. and statistical significance based on 3 biologically independent samples (each representing approximately 40 RPE fractions). Results are shown as fold change in RNA expression levels compared to the corresponding values from wild-type. Note that reduction in Pax6 gene dose in Pax6Sey-Neu/Pax6+;Mitfmi-ΔD/Mitfmi-ΔD mutants results in a 4–8 fold upregulation of retinal progenitor factors, whereas an increase in Pax6 gene dose in Pax6YAC/YAC;Mitfmi-ΔD/Mitfmi-ΔD mutants suppresses this upregulation. (TIF) [file pgen.1002757.s004.tif]

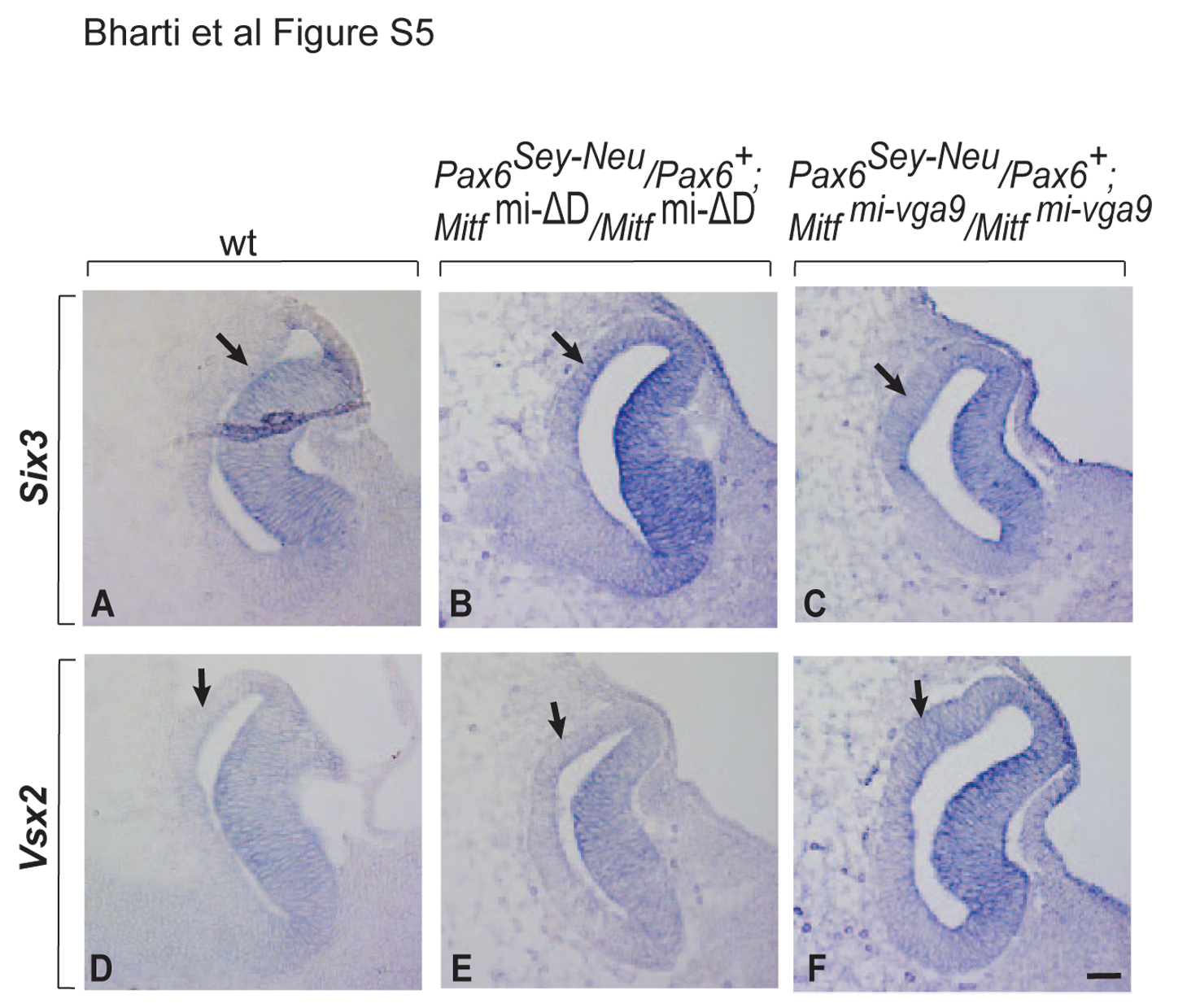

Supplement: Figure S5 — Ectopic expression of retinal progenitor transcription factors at the optic vesicle stage. (A–F) Eye sections from E10.0–E10.25 embryos of the indicated genotypes were subjected to in situ hybridization with the indicated probes. Arrows mark the RPE. Scale bar: 60 µm. (TIF) [file pgen.1002757.s005.tif]

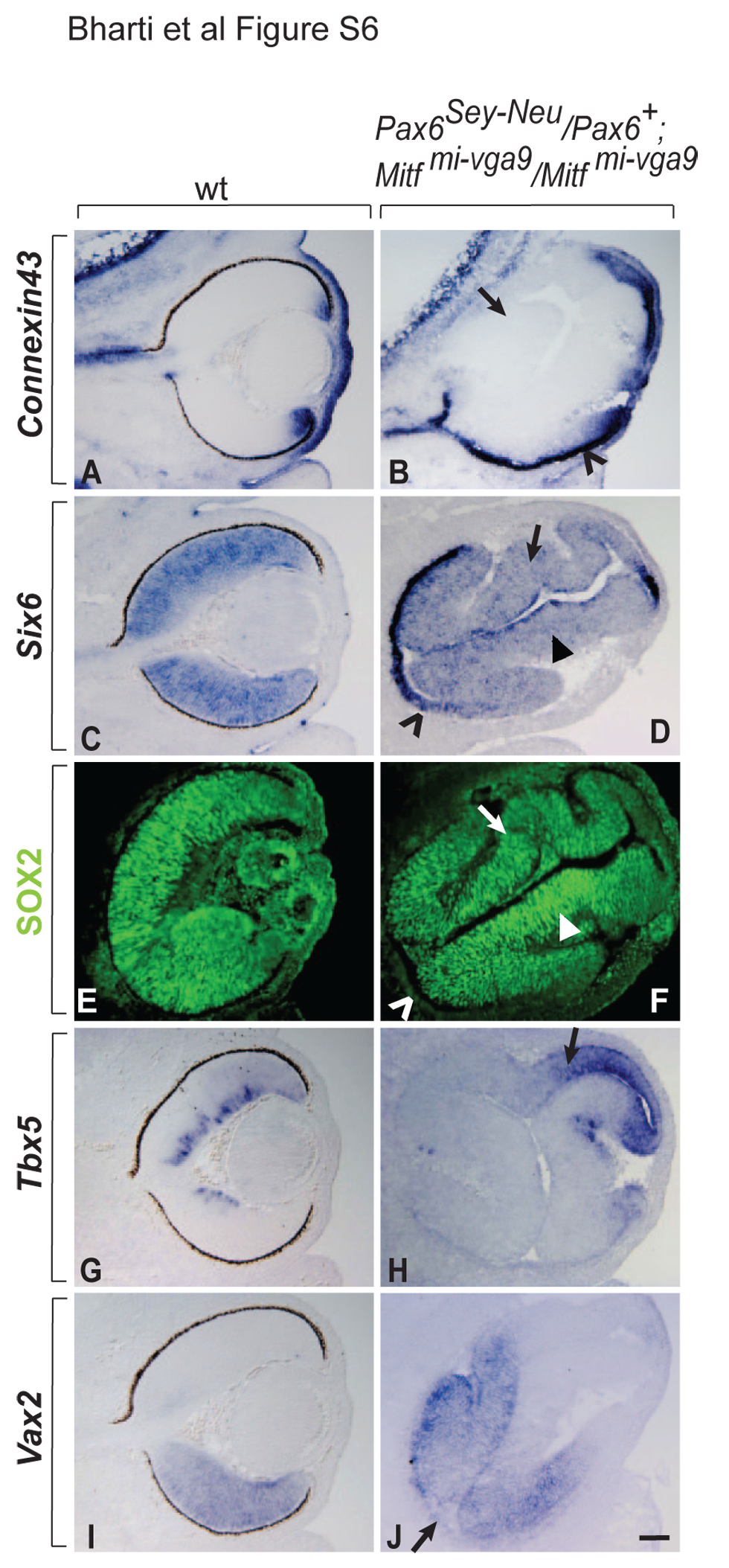

Supplement: Figure S6 — Only the dorsal RPE of E13.5 Pax6Sey-Neu/Pax6+;Mitfmi-vga9/Mitfmi-vga9 mutants shows transdifferentiation towards a second retina. (A,B) Expression of connexin 43, an RPE-marker, is affected only in the dorsal RPE of Pax6Sey-Neu/Pax6+;Mitfmi-vga9/Mitfmi-vga9 mutants. Arrow in (B) marks dorsal transdifferentiating portion, and open arrowhead ventral, non-transdifferentiating portion. (C–F) The retinal progenitor transcription factors Six6 and Sox2 are expressed in transdifferentiating dorsal RPE. In situ hybridization for Six6 or immunofluorescence for SOX2 show expression in the normal retina (solid arrowhead in D,F) and in transdifferentiated dorsal RPE (arrow in D,F). While Six6 expression can also be seen in non-transdifferentiated ventral RPE of double mutants (open arrowhead, D), SOX2 expression is absent from this region (open arrowhead, F). (G–J) Transdifferentiated RPE maintains its dorso-ventral polarity. In situ hybridization for Tbx5, a dorsal retina marker, and Vax2, a ventral retina marker, performed on E13.5 eye sections from Pax6/Mitf double mutants (arrows in H,J). Scale bar (A–D, G–J):115 µm; (E–F): 90 µm. (TIF) [file pgen.1002757.s006.tif]

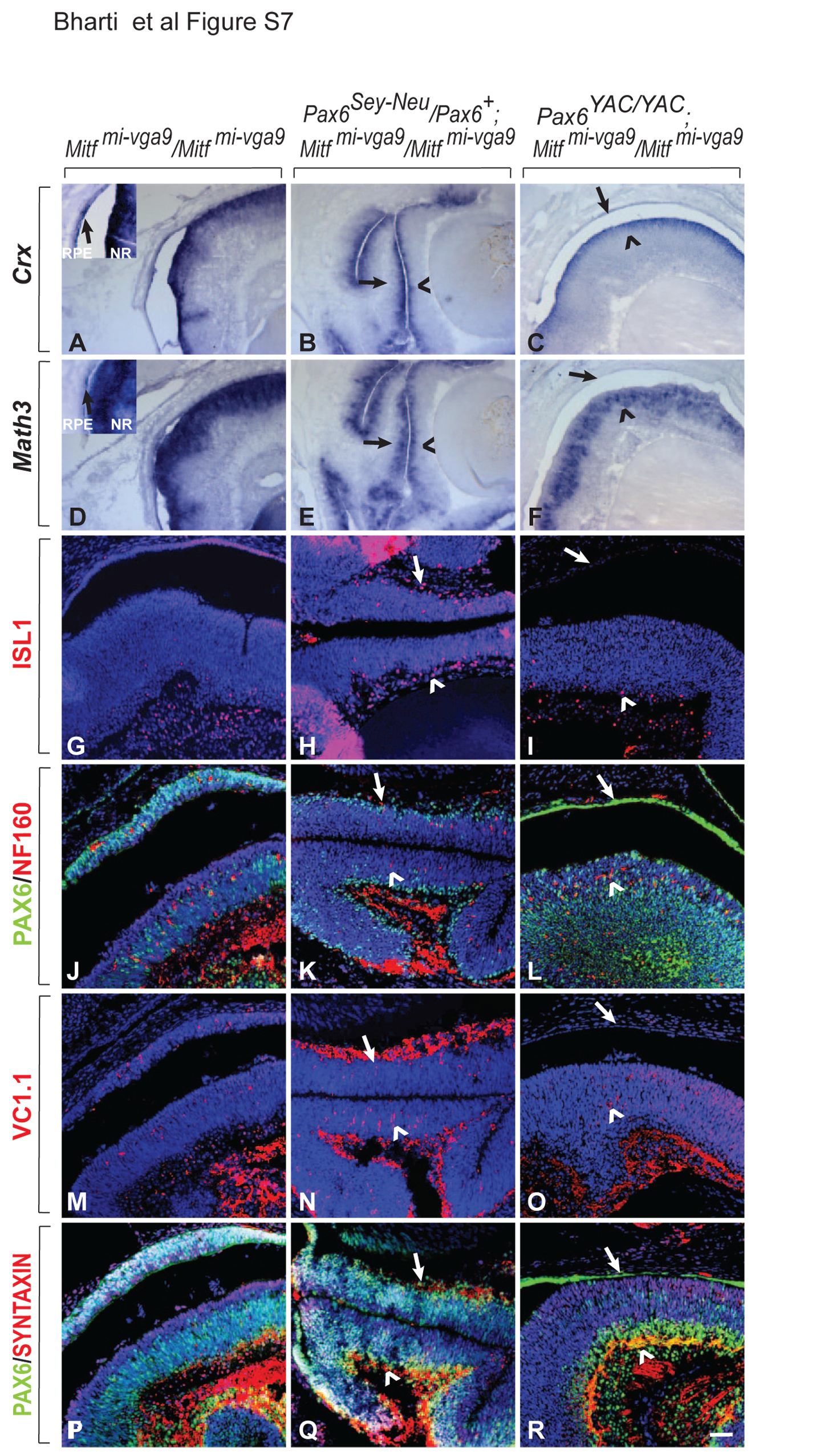

Supplement: Figure S7 — Development of a differentiated laminated retina in Pax6Sey-Neu/Pax6+;Mitfmi-vga9/Mitf mi-vga9 but not Pax6YAC/YAC;Mitf mi-vga9/Mitf mi-vga9 mice. Sections of eyes from P0 mice of the indicated genotypes were subjected to in situ hybridization for Crx, a photoreceptor marker (A–C) or Math3, an amacrine cell marker (D–F). Note that the RPE of Mitfmi-vga9/Mitfmi-vga9 mutants weakly expresses these two markers (see higher magnification of inset images) and ectopic staining is not present in the RPE of Pax6YAC/YAC;Mitfmi-vga9/Mitfmi-vga9 mutants (compare arrows in A,B and D,E with C,F for ectopic staining; arrowheads mark normal retinal staining). (G–R) Immunofluorescent labeling for the indicated markers on P0 eye sections of the indicated genotypes. ISL1 is a ganglion cell marker (G–I), as is PAX6 at this time point (J–L, P–R, green). NF160 marks horizontal cells (J–L, red); VC1.1 marks amacrine cells (M–O, red); and SYNTAXIN marks synapses (P–R, red). Arrows mark the transdifferentiating portions of the RPE in Pax6Sey-Neu/Pax6+;Mitfmi-vga9/Mitfmi-vga9 mice (H,K,N,Q) or the corresponding non-transdifferentiating portions in Pax6YAC/YAC;Mitf mi-vga9/Mitfmi-vga9 mice (I,L,O,R). The normal retinas continue to express each of these markers (arrowheads in the corresponding figures). Scale bar (A–F): 115 µm; (G–R): 90 µm. (TIF) [file pgen.1002757.s007.tif]

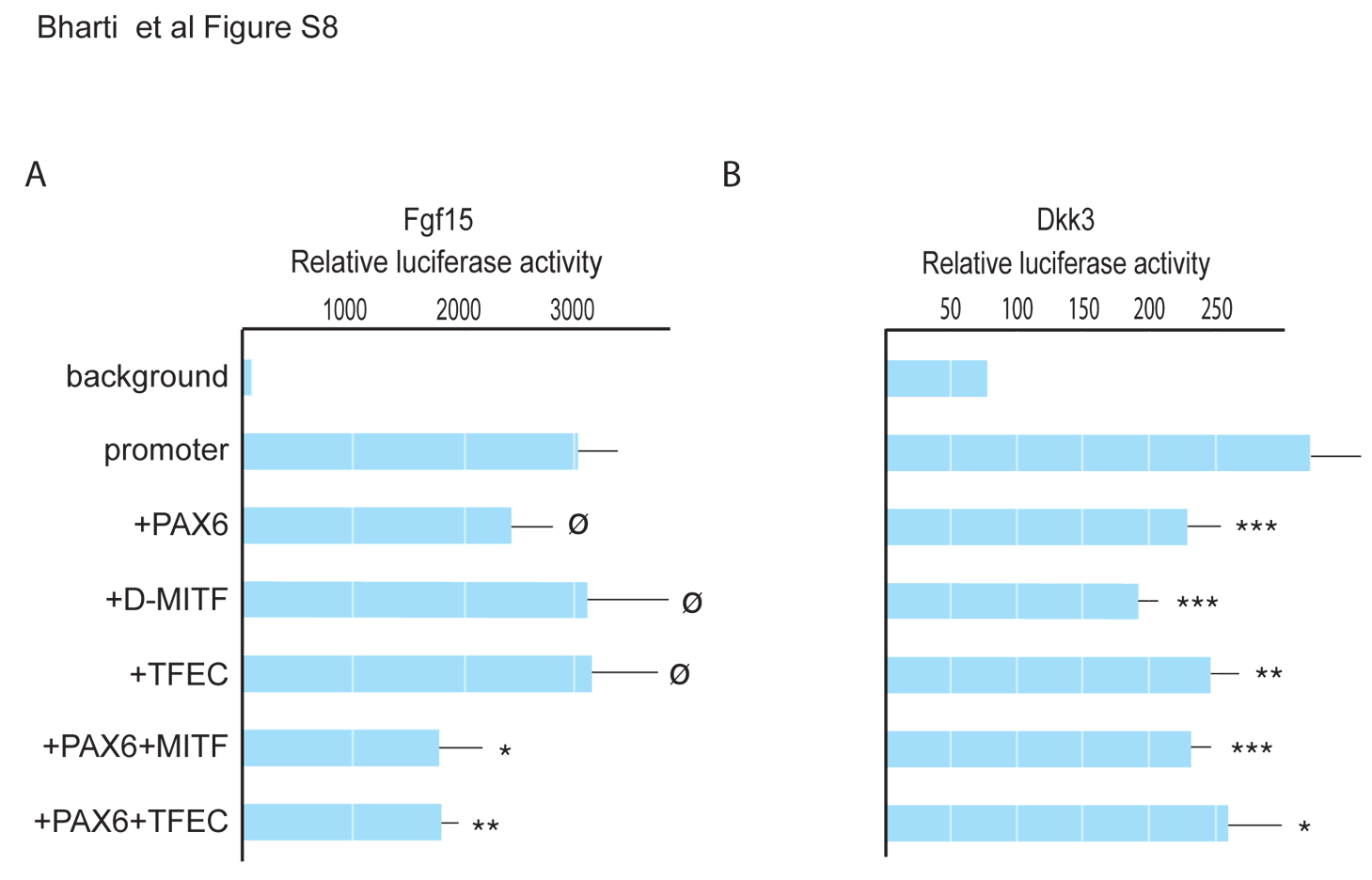

Supplement: Figure S8 — PAX6, MITF and TFEC regulate the activity of Fgf15 and Dkk3 promoter/enhancer regions in luciferase reporter assays. (A) A 1450 bp Fgf15 enhancer/promoter region was cloned in a vector containing the luciferase reporter and transfected into ARPE19 cells along with the indicated expression vectors. Each bar represents the mean luciferase activity units obtained from 8 independent transfections after normalization with a co-transfected control renilla luciferase construct. Error bars indicate S.D. and statistical significance is given for pairwise comparisons relative to promoter-only sample. (B) A 572 bp Dkk3 distal enhancer region was cloned upstream of 665 bp of the minimal Dkk3 promoter region and used as in (A). (TIF) [file pgen.1002757.s008.tif]
