# Supplementary material for: A Regulatory Loop Involving PAX6, MITF, and WNT Signaling Controls Retinal Pigment Epithelium Development
Source: PLoS Genet. 2012 Jul 5;8(7):e1002757. doi: 10.1371/journal.pgen.1002757 (PMC3390378; doi:10.1371/journal.pgen.1002757)
Supplement: Table S1 — Pax6 and Mitf alleles used in this study. Schematics of Pax6 and Mitf genomic loci, alleles used in this study, and a brief description of alleles is provided. (DOC) [file pgen.1002757.s009.doc]

| **Table S1: *Pax6* and *Mitf* alleles used in this study** | | | |
| --- | --- | --- | --- |
| **Gene** | **Genomic loci and alleles** | **Phenotype** | **References** |
| ***Pax6*** | ***Pax6*** genomic locus  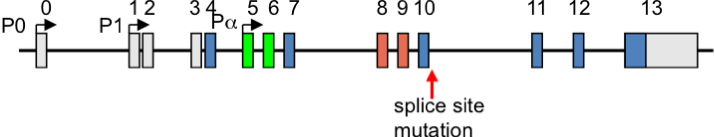 |  |  |
|  | ***Pax6^Sey-Neu^***: Splice site mutation, intron retention, stop codon after 23 amino acids, non-functional protein. | No visible effects on RPE pigmentation, no transdifferentiation. | Hill et al., 1991 |
|  | ***Pax6^YAC^****:* Transgene representing 5-6 copies of a 500 Mbp portion of the human PAX6 locus, leading to a 12-14-fold increase in Pax6 mRNA levels and a 2-3-fold increase in PAX6 protein levels when homozygous. Note that for this study, all *Pax6^Yac^* transgenics were homozygous for the transgene. | Slightly reduced/delayed RPE pigmentation. No RPE-transdifferentiation. | Schedl et al., 1996; Manuel et al., 2008 |
| ***Mitf*** | ***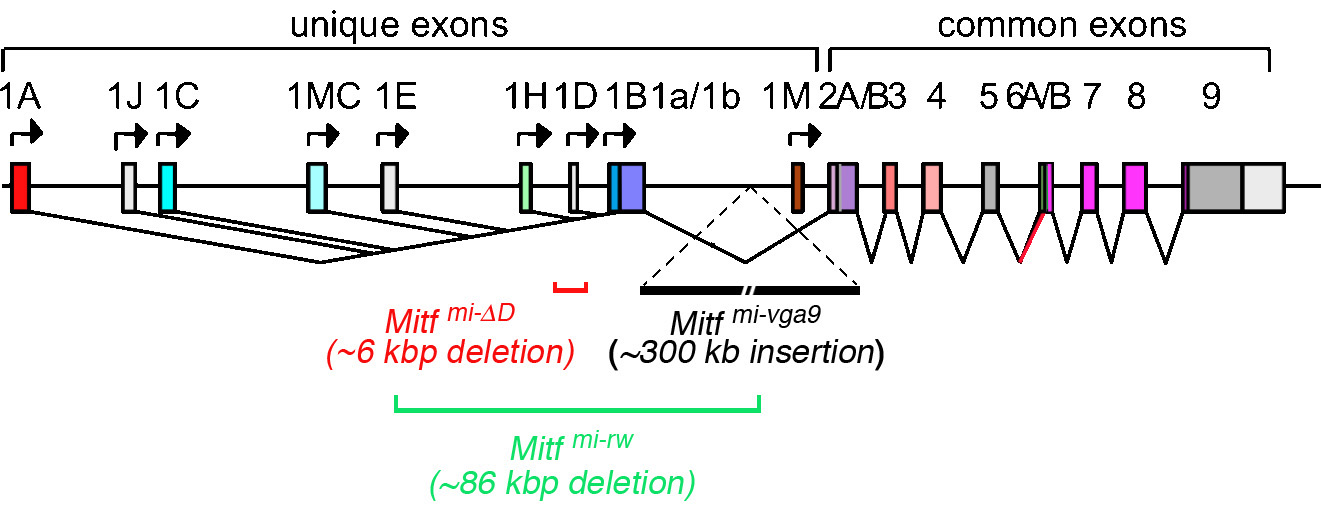***  ***Mitf*** genomic locus |  |  |
|  | ***Mitf  ^mi-^Δ^D^****:* Targeted deletion of D-isoform. RPE continues to express A- and H-Mitf isoforms. | Reduced/delayed pigmentation in the RPE. No RPE-retina trans-differentiation. | this manuscript |
|  | ***Mitf ^mi-vga9^***: Transgenic insertional mutant, no functional message or protein in the RPE. | No pigmentation in the RPE. RPE hyperproliferation and transdifferentiation of a small dorsal part of the RPE. | Hodgkinson et al., 1993 |
|  | ***Mitf ^mi-rw^***: Spontaneous mutant with missing expression of isoforms D and H. Low level expression of amino-terminally truncated MITF proteins initiated from internal start codons. | Severely reduced pigmentation in the RPE, present mostly in the distal RPE. Small part of the dorsal RPE transdifferentiates into a retina. | Bharti et al., 2008 |
