# Supplementary material for: A Regulatory Loop Involving PAX6, MITF, and WNT Signaling Controls Retinal Pigment Epithelium Development
Source: PLoS Genet. 2012 Jul 5;8(7):e1002757. doi: 10.1371/journal.pgen.1002757 (PMC3390378; doi:10.1371/journal.pgen.1002757)
Supplement: Table S2 — Affymetrix microarray and bioinformatics analysis. RNA was prepared from RPE-fractions from three biological replicates each for wild type and the different Pax6/Mitf mutants and checked for integrity using bioanalyzer. It was then used for hybridization with Affymetrix Mouse Gene 1.0 ST Chip. Raw data were processed using Robust Multi-Array and analyzed using Genespring 7.0 software (Agilent, Santa Clara, CA). Data were normalized to the statistical mean of all detectable probe sets and its statistical significance was tested by ANOVA analysis. A total of 532 gene fragments were selected based on a maximum coefficient of variation of 1.5 and turkey p-values less than 0.05. (DOCX) [file pgen.1002757.s010.docx]

| **Table S2: Gene expression profiles in mutant E11.5 RPE fractions**  **Genes upregulated in *Pax6^Sey-Neu^/Pax6^+^;Mitf^mi-ΔD^/Mitf^mi-ΔD^* as compared to wild type (fold change)** | | | | | | | |
| --- | --- | --- | --- | --- | --- | --- | --- |
| **Gene name** | **Gene Symbol** | **Gene ID** | ***Pax6^Sey-Neu^/***  ***Pax6^+^*** | ***Mitf^mi-ΔD^/***  ***Mitf^mi-ΔD^*** | ***Pax6^Sey-Neu^/***  ***Pax6^+^;***  ***Mitf^mi-ΔD^/***  ***Mitf^mi-ΔD^*** | ***Pax6^YAC/YAC^; Mitf^mi-ΔD^/***  ***Mitf^mi-ΔD^*** | ***Pax6^YAC/YAC^*** |
| carboxypeptidase A3 (mast cell) | *Cpa3* | 12873 | 1.27 | 1.18 | 5.90 | 1.11 | -1.09 |
| fibroblast growth factor 15 | *Fgf15* | 14170 | 1.06 | -1.04 | 5.17 | 1.01 | 1.16 |
| corticotropin releasing hormone binding protein | *Crhbp* | 12919 | -1.12 | -1.14 | 5.15 | 1.12 | 1.23 |
| aldehyde dehydrogenase 1 family, member A1 | *Aldh1a1* | 11668 | -1.45 | -1.49 | 4.79 | -1.95 | -1.38 |
| myosin, heavy chain 3, skeletal muscle, embryonic | *Myh3* | 17883 | 1.52 | 1.71 | 4.26 | 1.53 | -1.06 |
| collagen, type XIV, alpha 1 | *Col14a1* | 12818 | -1.06 | -1.10 | 4.26 | 1.43 | 1.45 |
| STEAP family member 4 | *Steap4* | 117167 | 1.02 | 1.08 | 3.96 | 1.15 | 1.30 |
| visual system homeobox 2 | *Vsx2* | 12677 | -1.41 | -1.25 | 3.83 | 1.13 | 1.28 |
| carboxypeptidase X (M14 family), member 2 | *Cpxm2* | 55987 | -1.02 | -1.19 | 3.78 | 1.40 | 1.38 |
| retina and anterior neural fold homeobox | *Rax* | 19434 | -1.04 | -1.11 | 3.62 | 1.13 | 1.23 |
| keratocan | *Kera* | 16545 | 1.05 | 1.08 | 3.51 | 1.29 | -1.04 |
| decorin | *Dcn* | 13179 | -1.06 | -1.20 | 3.51 | -1.09 | 1.29 |
| T-box 20 | *Tbx20* | 57246 | 1.04 | -1.12 | 3.26 | 1.11 | -1.07 |
| solute carrier family 7, (cationic amino acid transporter, y+ system) member 11 | *Slc7a11* | 26570 | 1.40 | 2.53 | 3.17 | 1.70 | -1.09 |
| collagen, type I, alpha 1 | *Col1a1* | 12842 | 1.01 | 1.05 | 3.12 | 1.41 | 1.41 |
| WAP four-disulfide core domain 1 | *Wfdc1* | 67866 | -1.14 | -1.14 | 3.10 | -1.29 | -1.29 |
| paired box 6 | *Pax6* | 18508 | 1.63 | 1.07 | 3.03 | 1.24 | 1.04 |
| Zic family member 1 (odd-paired homolog, Drosophila) | *Zic1* | 22771 | 1.63 | 1.10 | 2.93 | 1.30 | 1.14 |
| collagen, type VI, alpha 1 | *Col6a1* | 12833 | 1.06 | 1.12 | 2.83 | 1.28 | 1.27 |
| aquaporin 1 (Colton blood group) | *Aqp1* | 11826 | -1.10 | -1.03 | 2.71 | 1.10 | 1.12 |
| actin, alpha, cardiac muscle 1 | *Actc1* | 11464 | 1.33 | 1.81 | 2.71 | 1.54 | -1.11 |
| SPARC related modular calcium binding 2 | *Smoc2* | 64074 | -1.03 | 1.04 | 2.62 | 1.07 | -1.01 |
| claudin 1 | *Cldn1* | 12737 | -1.27 | -1.19 | 2.61 | -1.02 | -1.03 |
| dickkopf homolog 3 (Xenopus laevis) | *Dkk3* | 50781 | -1.15 | -1.08 | 2.60 | 1.25 | 1.19 |
| T-box 22 | *Tbx22* | 245572 | 1.02 | 1.48 | 2.49 | 1.59 | 1.07 |
| ST8 alpha-N-acetyl-neuraminide alpha-2,8-sialyltransferase 1 | *St8sia1* | 20449 | 1.08 | -1.09 | 2.49 | 1.10 | 1.03 |
| SIX homeobox 6 | *Six6* | 20476 | -1.12 | 1.00 | 2.44 | 1.57 | 1.38 |
| collagen, type VI, alpha 3 | *Col6a3* | 12835 | -1.05 | 1.06 | 2.40 | 1.44 | 1.22 |
| myosin, light chain 4, alkali; atrial, embryonic | *Myl4* | 17896 | 1.44 | 1.37 | 2.34 | 1.44 | 1.10 |
| Zic family member 5 (odd-paired homolog, Drosophila) | *Zic5* | 65100 | 1.40 | 1.10 | 2.34 | 1.52 | 1.25 |
| keratin 5 | *Krt5* | 110308 | -1.02 | 1.02 | 2.27 | -1.15 | -1.10 |
| RAS-like, estrogen-regulated, growth inhibitor | *Rerg* | 232441 | 1.07 | 1.20 | 2.26 | 1.21 | 1.15 |
| biglycan | *Bgn* | 12111 | 1.08 | 1.07 | 2.25 | 1.21 | 1.15 |
| troponin C type 1 (slow) | *Tnnc1* | 21924 | 1.27 | 1.25 | 2.24 | 1.43 | 1.12 |
| annexin A1 | *Anxa1* | 16952 | -1.09 | 1.03 | 2.21 | -1.47 | -1.53 |
| cathepsin K | *Ctsk* | 13038 | 1.06 | -1.14 | 2.20 | 1.10 | 1.07 |
| thrombospondin 2 | *Thbs2* | 21826 | -1.08 | -1.17 | 2.18 | -1.19 | -1.14 |
| titin | *Ttn* | 22138 | 1.19 | 1.55 | 2.16 | 1.32 | -1.12 |
| aldehyde dehydrogenase 1 family, member A2 | *Aldh1a2* | 19378 | -1.03 | 1.14 | 2.14 | 1.34 | 1.22 |
| collagen, type XII, alpha 1 | *Col12a1* | 12816 | 1.08 | -1.05 | 2.13 | -1.12 | -1.03 |
| Zic family member 2 (odd-paired homolog, Drosophila) | *Zic2* | 22772 | 1.31 | 1.03 | 2.10 | 1.62 | 1.34 |
| leucine rich repeat containing 17 | *Lrrc17* | 74511 | -1.14 | 1.01 | 2.10 | 1.28 | 1.16 |
| collagen, type V, alpha 2 | *Col5a2* | 12832 | -1.02 | 1.07 | 2.09 | 1.16 | 1.27 |
| RAR-related orphan receptor B | *Rorb* | 225998 | -1.24 | -1.07 | 2.07 | 1.14 | 1.14 |
| collagen, type VI, alpha 2 | *Col6a2* | 12834 | -1.02 | 1.01 | 2.05 | 1.29 | 1.23 |
| matrilin 4 | *Matn4* | 17183 | -1.02 | -1.01 | 2.05 | -1.01 | 1.04 |
| mab-21-like 2 (C. elegans) | *Mab21l2* | 23937 | 1.14 | 1.22 | 2.04 | 1.31 | -1.01 |
| desmocollin 3 | *Dsc3* | 13507 | 1.18 | -1.07 | 2.04 | -1.10 | -1.09 |
| CD44 molecule (Indian blood group) | *Cd44* | 12505 | 1.10 | 1.12 | 2.04 | 1.16 | -1.00 |
| leucine rich repeat neuronal 1 | *Lrrn1* | 16979 | 1.25 | 1.20 | 2.03 | 1.26 | -1.14 |
| integral membrane protein 2A | *Itm2a* | 16431 | -1.07 | 1.09 | 2.01 | 1.22 | 1.12 |
| SRY (sex determining region Y)-box 2 | *Sox2* | 20674 | 1.32 | -1.05 | 1.97 | -1.05 | 1.03 |
| EGF-like-domain, multiple 6 | *Egfl6* | 54156 | -1.15 | -1.05 | 1.97 | 1.25 | 1.19 |
| C1q and tumor necrosis factor related protein 7 | *C1qtnf7* | 109323 | 1.11 | 1.21 | 1.96 | 1.43 | 1.33 |
| keratin 15 | *Krt15* | 16665 | 1.03 | -1.13 | 1.96 | 1.20 | 1.18 |
| Cdon homolog (mouse) | *Cdon* | 57810 | -1.02 | 1.02 | 1.94 | 1.04 | 1.04 |
| lysyl oxidase | *Lox* | 16948 | 1.05 | 1.01 | 1.92 | 1.00 | -1.13 |
| sema domain, immunoglobulin domain (Ig), short basic domain, secreted, (semaphorin) 3C | *Sema3c* | 20348 | 1.16 | -1.04 | 1.92 | 1.06 | 1.07 |
| glycine dehydrogenase (decarboxylating) | *Gldc* | 104174 | 1.12 | -1.12 | 1.91 | -1.07 | -1.18 |
| collagen, type III, alpha 1 | *Col3a1* | 12825 | -1.02 | 1.08 | 1.89 | 1.19 | 1.08 |
| DNA-damage-inducible transcript 4-like | *Ddit4l* | 73284 | 1.11 | 1.00 | 1.89 | 1.10 | 1.11 |
| ets variant 1 | *Etv1* | 14009 | -1.05 | 1.14 | 1.87 | 1.07 | -1.05 |
| PDZ and LIM domain 3 | *Pdlim3* | 53318 | -1.04 | 1.26 | 1.85 | 1.35 | 1.07 |
| filamin C, gamma (actin binding protein 280) | *Flnc* | 68794 | 1.03 | 1.19 | 1.84 | 1.36 | 1.11 |
| KDEL (Lys-Asp-Glu-Leu) endoplasmic reticulum protein retention receptor 3 | *Kdelr3* | 105785 | 1.00 | -1.08 | 1.83 | -1.17 | -1.13 |
| fibulin 5 | *Fbln5* | 23876 | 1.06 | 1.14 | 1.83 | 1.09 | 1.03 |
| nuclear factor I/X (CCAAT-binding transcription factor) | *Nfix* | 18032 | -1.02 | 1.08 | 1.83 | 1.16 | -1.02 |
| keratin 19 | *Krt19* | 16669 | -1.03 | -1.19 | 1.82 | -1.06 | -1.03 |
| latent transforming growth factor beta binding protein 1 | *Ltbp1* | 268977 | -1.09 | -1.01 | 1.82 | 1.35 | 1.16 |
| protease, serine, 35 | *Prss35* | 244954 | -1.10 | -1.01 | 1.82 | 1.27 | 1.04 |
| actin, alpha 1, skeletal muscle | *Acta1* | 11459 | 1.35 | 1.30 | 1.81 | 1.49 | 1.22 |
| integrin, beta 8 | *Itgb8* | 320910 | 1.18 | 1.06 | 1.80 | 1.03 | 1.04 |
| small nucleolar RNA, C/D box 22 | *Snord22* | 100127111 | -1.22 | 1.20 | 1.79 | 1.47 | 1.10 |
| v-myb myeloblastosis viral oncogene homolog (avian) | *Myb* | 17863 | -1.17 | -1.01 | 1.78 | -1.02 | -1.01 |
| ADAM metallopeptidase with thrombospondin type 1 motif, 9 | *Adamts9* | 101401 | -1.01 | 1.07 | 1.78 | 1.15 | 1.01 |
| growth hormone receptor | *Ghr* | 14600 | 1.02 | 1.02 | 1.77 | 1.04 | -1.03 |
| collagen, type I, alpha 2 | *Col1a2* | 12843 | 1.01 | 1.05 | 1.75 | 1.23 | 1.15 |
| ADAM metallopeptidase with thrombospondin type 1 motif, 9 | *Adamts9* | 101401 | -1.04 | 1.05 | 1.75 | 1.14 | 1.01 |
| cAMP responsive element binding protein 3-like 1 | *Creb3l1* | 26427 | -1.02 | -1.01 | 1.75 | 1.04 | 1.01 |
| dickkopf homolog 1 (Xenopus laevis) | *Dkk1* | 13380 | 1.07 | 1.22 | 1.74 | 2.31 | 1.57 |
| nuclear factor I/B | *Nfib* | 18028 | 1.08 | 1.15 | 1.72 | 1.17 | 1.06 |
| hyperpolarization activated cyclic nucleotide-gated potassium channel 1 | *Hcn1* | 15165 | -1.07 | 1.43 | 1.72 | 2.83 | 2.15 |
| ras homolog gene family, member J | *Rhoj* | 80837 | -1.01 | -1.02 | 1.72 | 1.00 | -1.03 |
| actinin, alpha 2 | *Actn2* | 11472 | 1.17 | 1.12 | 1.70 | 1.15 | -1.11 |
| troponin I type 1 (skeletal, slow) | *Tnni1* | 21952 | 1.29 | 1.30 | 1.70 | 1.34 | 1.11 |
| scleraxis homolog B (mouse) | *Scxb* | 20289 | 1.07 | 1.03 | 1.70 | 1.14 | 1.08 |
| periostin, osteoblast specific factor | *Postn* | 50706 | 1.23 | 1.16 | 1.69 | -1.62 | -1.82 |
| N-terminal EF-hand calcium binding protein 2 | *Necab2* | 117148 | -1.02 | -1.01 | 1.69 | 1.18 | 1.09 |
| mannose receptor, C type 1 | *Mrc1* | 17533 | -1.05 | -1.14 | 1.69 | 1.06 | 1.07 |
| Nik related kinase | *Nrk* | 27206 | 1.03 | 1.22 | 1.68 | 1.19 | -1.02 |
| UDP-N-acetyl-alpha-D-galactosamine:polypeptide N-acetylgalactosaminyltransferase-like 1 | *Galntl1* | 108760 | 1.02 | -1.02 | 1.67 | 1.04 | -1.03 |
| poliovirus receptor-related 3 | *Pvrl3* | 58998 | 1.05 | 1.13 | 1.67 | 1.10 | 1.06 |
| epithelial membrane protein 1 | *Emp1* | 13730 | 1.01 | 1.02 | 1.66 | 1.31 | 1.16 |
| echinoderm microtubule associated protein like 5 | *Eml5* | 319670 | -1.12 | -1.04 | 1.66 | 1.30 | 1.20 |
| pleiotrophin | *Ptn* | 19242 | 1.29 | 1.13 | 1.66 | 1.11 | 1.14 |
| plexin C1 | *Plxnc1* | 54712 | 1.08 | -1.11 | 1.65 | 1.14 | 1.13 |
| tenascin C | *Tnc* | 21923 | -1.16 | 1.14 | 1.65 | 1.10 | -1.04 |
| creatine kinase, brain | *Ckb* | 12709 | 1.56 | -1.07 | 1.64 | 1.13 | 1.08 |
| HtrA serine peptidase 1 | *Htra1* | 56213 | 1.14 | -1.22 | 1.63 | -1.11 | -1.12 |
| hypermethylated in cancer 1 | *Hic1* | 15248 | 1.02 | 1.05 | 1.62 | 1.29 | 1.17 |
| collagen, type IX, alpha 1 | *Col9a1* | 12839 | -1.00 | 1.02 | 1.61 | 1.16 | 1.03 |
| ets variant 1 | *Etv1* | 14009 | -1.06 | 1.14 | 1.61 | 1.03 | -1.09 |
| anoctamin 1, calcium activated chloride channel | *Ano1* | 101772 | -1.00 | -1.21 | 1.61 | -1.78 | -1.54 |
| SH3 domain binding glutamic acid-rich protein like 2 | *Sh3bgrl2* | 212531 | 1.02 | 1.07 | 1.61 | 1.22 | 1.17 |
| ADAM metallopeptidase with thrombospondin type 1 motif, 9 | *Adamts9* | 101401 | -1.05 | 1.04 | 1.60 | 1.11 | -1.02 |
| elastin | *Eln* | 13717 | 1.03 | 1.09 | 1.60 | 1.05 | -1.10 |
| angiopoietin-like 1 | *Angptl1* | 72713 | -1.03 | 1.14 | 1.60 | -1.00 | -1.05 |
| myosin, light polypeptide 9, regulatory | *Myl9* | 98932 | 1.05 | 1.11 | 1.59 | -1.01 | 1.05 |
| nuclear factor I/B | *Nfib* | 18028 | 1.00 | 1.06 | 1.59 | 1.03 | -1.02 |
| angiotensin II receptor, type 2 | *Agtr2* | 11609 | 1.06 | -1.12 | 1.59 | -1.14 | -1.22 |
| collagen, type XVII, alpha 1 | *Col17a1* | 12821 | -1.01 | 1.06 | 1.58 | 1.30 | 1.28 |
| CUG triplet repeat, RNA binding protein 2 | *Cugbp2* | 14007 | 1.18 | 1.05 | 1.58 | 1.15 | 1.03 |
| flavin containing monooxygenase 1 | *Fmo1* | 14261 | 1.01 | -1.11 | 1.58 | 1.04 | 1.04 |
| lymphocyte-specific protein 1 | *Lsp1* | 16985 | 1.07 | 1.14 | 1.58 | 1.19 | 1.19 |
| Rho GTPase activating protein 24 | *Arhgap24* | 231532 | -1.00 | 1.09 | 1.58 | 1.12 | 1.09 |
| Meis homeobox 1 | *Meis1* | 17268 | 1.07 | 1.06 | 1.57 | 1.38 | 1.20 |
| nuclear receptor subfamily 3, group C, member 1 (glucocorticoid receptor) | *Nr3c1* | 14815 | -1.04 | -1.14 | 1.57 | 1.00 | -1.00 |
| IQ motif containing GTPase activating protein 2 | *Iqgap2* | 544963 | 1.00 | -1.07 | 1.57 | -1.17 | -1.02 |
| solute carrier family 40 (iron-regulated transporter), member 1 | *Slc40a1* | 53945 | 1.13 | 1.11 | 1.57 | 1.08 | 1.12 |
| collagen, type V, alpha 1 | *Col5a1* | 12831 | -1.00 | -1.00 | 1.57 | 1.17 | 1.10 |
| delta-like 1 homolog (Drosophila) | *Dlk1* | 13386 | -1.16 | 1.10 | 1.57 | 1.14 | 1.11 |
| ecotropic viral integration site 1 | *Evi1* | 14013 | -1.20 | -1.10 | 1.56 | 1.05 | -1.03 |
| cysteine dioxygenase, type I | *Cdo1* | 12583 | 1.05 | 1.10 | 1.55 | 1.23 | 1.23 |
| protein phosphatase 1, regulatory (inhibitor) subunit 12B | *Ppp1r12b* | 329251 | 1.11 | 1.07 | 1.55 | 1.36 | 1.34 |
| protease, serine, 12 (neurotrypsin, motopsin) | *Prss12* | 19142 | 1.02 | 1.09 | 1.55 | 1.11 | 1.03 |
| frizzled homolog 5 (Drosophila) | *Fzd5* | 14367 | -1.09 | -1.09 | 1.55 | 1.11 | 1.04 |
| catenin (cadherin-associated protein), delta 2 (neural plakophilin-related arm-repeat protein) | *Ctnnd2* | 18163 | -1.16 | -1.06 | 1.55 | 1.35 | 1.23 |
| mesenchyme homeobox 1 | *Meox1* | 17285 | -1.08 | 1.17 | 1.54 | 1.06 | 1.02 |
| glypican 4 | *Gpc4* | 14735 | 1.01 | 1.07 | 1.54 | 1.02 | 1.00 |
| glycine amidinotransferase (L-arginine:glycine amidinotransferase) | *Gatm* | 67092 | 1.15 | 1.10 | 1.52 | 1.03 | 1.23 |
| slit homolog 2 (Drosophila) | *Slit2* | 20563 | 1.10 | 1.04 | 1.51 | 1.39 | 1.24 |
| secernin 1 | *Scrn1* | 69938 | 1.21 | 1.22 | 1.51 | 1.34 | 1.25 |
| T-box 3 | *Tbx3* | 21386 | 1.01 | 1.03 | 1.51 | 1.04 | 1.04 |
| synaptotagmin XI | *Syt11* | 229521 | 1.25 | -1.13 | 1.51 | 1.04 | -1.03 |
| calcium channel, voltage-dependent, L type, alpha 1D subunit | *Cacna1d* | 12289 | -1.17 | -1.11 | 1.51 | -1.03 | -1.05 |
| unc-13 homolog B (C. elegans) | *Unc13b* | 22249 | 1.15 | 1.10 | 1.51 | 1.22 | 1.15 |
| mab-21-like 1 (C. elegans) | *Mab21l1* | 17116 | 1.04 | -1.10 | 1.50 | -1.02 | -1.11 |
| transcription factor EC | *Tfec* | 21426 | -1.15 | 1.87 | 1.49 | 2.64 | 1.71 |
| V-set and transmembrane domain containing 2A | *Vstm2a* | 211739 | 1.15 | 1.82 | 1.49 | 3.28 | 2.05 |
| lymphocyte antigen 86 | *Ly86* | 17084 | 1.13 | -1.04 | 1.49 | -1.20 | -1.07 |
| protocadherin 7 | *Pcdh7* | 54216 | -1.02 | 1.03 | 1.49 | 1.02 | 1.01 |
| sushi, von Willebrand factor type A, EGF and pentraxin domain containing 1 | *Svep1* | 64817 | -1.04 | 1.05 | 1.49 | 1.04 | -1.05 |
| phosphodiesterase 7B | *Pde7b* | 29863 | -1.02 | 1.08 | 1.49 | 1.13 | 1.12 |
| cadherin 4, type 1, R-cadherin (retinal) | *Cdh4* | 12561 | -1.05 | -1.09 | 1.49 | 1.34 | 1.30 |
| ankyrin 2, neuronal | *Ank2* | 109676 | 1.07 | -1.03 | 1.49 | 1.04 | -1.01 |
|  |  |  |  |  |  |  |  |
|  |  |  |  |  |  |  |  |
|  |  |  |  |  |  |  |  |
| **Genes downregulated in *Pax6^Sey-Neu^/Pax6^+^;Mitf^mi-ΔD^/Mitf^mi-ΔD^* as compared to wild type (fold change)** | | | | | | | |
| **Gene name** | **Gene Symbol** | **Gene ID** | ***Pax6^Sey-Neu^/***  ***Pax6^+^*** | ***Mitf ^mi-ΔD^/***  ***Mitf ^mi-ΔD^*** | ***Pax6^Sey-Neu^/***  ***Pax6^+^;***  ***Mitf ^mi-ΔD^/***  ***Mitf ^mi-ΔD^*** | ***Pax6^YAC/YAC^; Mitf ^mi-ΔD^/***  ***Mitf ^mi-ΔD^*** | ***Pax6^YAC/YAC^*** |
| glycoprotein (transmembrane) nmb | *Gpnmb* | 93695 | -1.33 | -1.87 | -2.98 | -1.35 | 1.32 |
| G protein-coupled receptor 143 | *Gpr143* | 18241 | -1.19 | -2.32 | -2.97 | -2.58 | -1.00 |
| S100 calcium binding protein A1 | *S100a1* | 20193 | -1.12 | -1.72 | -2.47 | -2.16 | 1.16 |
| beta-site APP-cleaving enzyme 2 | *Bace2* | 56175 | -1.13 | -1.85 | -2.34 | -1.96 | 1.06 |
| crystallin, alpha A | *Cryaa* | 12954 | -2.79 | -2.15 | -2.32 | -1.89 | -1.17 |
| pro-platelet basic protein (chemokine (C-X-C motif) ligand 7) | *Ppbp* | 57349 | -1.03 | -1.27 | -2.21 | -1.56 | -1.76 |
| retinaldehyde binding protein 1 | *Rlbp1* | 19771 | -1.00 | -1.67 | -2.16 | -2.06 | -1.24 |
| solute carrier family 45, member 2 | *Slc45a2* | 22293 | -1.22 | -1.40 | -2.02 | -1.67 | -1.11 |
| solute carrier family 11 (proton-coupled divalent metal ion transporters), member 1 | *Slc11a1* | 18173 | -1.15 | -1.54 | -2.02 | -1.28 | 1.10 |
| hemoglobin, zeta | *Hbz* | 15126 | -1.17 | -1.08 | -1.96 | -1.18 | -1.03 |
| solute carrier family 4, anion exchanger, member 1 (erythrocyte membrane protein band 3, Diego blood group) | *Slc4a1* | 20533 | -1.20 | -1.06 | -1.91 | -1.33 | -1.21 |
| monoglyceride lipase | *Mgll* | 23945 | -1.12 | -1.39 | -1.90 | -1.43 | 1.15 |
| hydroxy-delta-5-steroid dehydrogenase, 3 beta- and steroid delta-isomerase 1 | *Hsd3b1* | 15492 | 1.10 | -1.13 | -1.86 | -1.03 | -1.04 |
| optineurin | *Optn* | 71648 | -1.11 | -1.56 | -1.84 | -1.67 | 1.01 |
| tyrosinase-related protein 1 | *Tyrp1* | 22178 | -1.04 | -1.48 | -1.82 | -1.51 | 1.10 |
| RAB27A, member RAS oncogene family | *Rab27a* | 11891 | -1.12 | -1.40 | -1.81 | -1.09 | 1.28 |
| melan-A | *Mlana* | 77836 | -1.11 | -1.79 | -1.72 | -1.57 | 1.30 |
| transthyretin | *Ttr* | 22139 | 1.55 | -2.85 | -1.72 | -2.97 | -2.87 |
| silver homolog (mouse) | *Silv* | 20431 | -1.03 | -1.31 | -1.70 | -1.25 | 1.06 |
| guanosine monophosphate reductase | *Gmpr* | 66355 | -1.05 | -1.39 | -1.69 | -1.32 | 1.03 |
| synaptotagmin-like 2 | *Sytl2* | 83671 | 1.00 | -1.52 | -1.69 | -2.30 | -1.59 |
| Dmx-like 2 | *Dmxl2* | 235380 | -1.10 | -1.38 | -1.68 | -1.51 | -1.08 |
| platelet factor 4 | *Pf4* | 56744 | -1.14 | -1.42 | -1.68 | -1.62 | -1.55 |
| carbonic anhydrase II | *Ca2* | 12349 | -1.10 | 1.01 | -1.67 | -1.30 | -1.29 |
| junctophilin 1 | *Jph1* | 57339 | -1.18 | -1.16 | -1.67 | -1.48 | -1.34 |
| solute carrier family 7 (cationic amino acid transporter, y+ system), member 8 | *Slc7a8* | 50934 | -1.21 | -1.39 | -1.66 | -1.73 | 1.13 |
| tetraspanin 10 | *Tspan10* | 208634 | -1.11 | -1.42 | -1.65 | -1.17 | 1.12 |
| epoxide hydrolase 1, microsomal (xenobiotic) | *Ephx1* | 13849 | -1.02 | -1.51 | -1.63 | -2.33 | -1.32 |
| tyrosinase (oculocutaneous albinism IA) | *Tyr* | 22173 | -1.04 | -1.20 | -1.62 | -1.55 | 1.02 |
| complement component 1, q subcomponent-like 3 | *C1ql3* | 227580 | 1.10 | -1.32 | -1.61 | -2.41 | -1.55 |
| retinol dehydrogenase 5 (11-cis/9-cis) | *Rdh5* | 19682 | 1.06 | -1.32 | -1.61 | -1.64 | -1.25 |
| SRY (sex determining region Y)-box 9 | *Sox9* | 20682 | 1.03 | -1.12 | -1.60 | -1.57 | -1.38 |
| forkhead box F2 | *Foxf2* | 14238 | -1.00 | -1.21 | -1.60 | -2.03 | -1.51 |
| SIX homeobox 2 | *Six2* | 20472 | -1.16 | 1.04 | -1.60 | -1.29 | -1.14 |
| solute carrier family 24, member 5 | *Slc24a5* | 317750 | -1.05 | -1.50 | -1.59 | -1.75 | -1.07 |
| Dmx-like 2 | *Dmxl2* | 235380 | -1.05 | -1.30 | -1.58 | -1.48 | -1.06 |
| zinc finger, DHHC-type containing 2 | *Zdhhc2* | 70546 | -1.00 | -1.25 | -1.58 | -1.24 | 1.07 |
| vitrin | *Vit* | 74199 | 1.07 | -1.27 | -1.58 | -2.35 | -2.07 |
| forkhead box C2 (MFH-1, mesenchyme forkhead 1) | *Foxc2* | 14234 | -1.09 | -1.06 | -1.57 | -1.31 | -1.16 |
| forkhead box L1 | *Foxl1* | 14241 | -1.10 | -1.09 | -1.57 | -1.41 | -1.29 |
| phospholipid transfer protein | *Pltp* | 18830 | -1.13 | -1.33 | -1.56 | -1.33 | -1.08 |
| dipeptidyl-peptidase 4 | *Dpp4* | 13482 | -1.19 | -1.44 | -1.56 | -1.90 | -1.16 |
| acid phosphatase 5, tartrate resistant | *Acp5* | 11433 | -1.10 | -1.08 | -1.54 | -1.26 | -1.21 |
| oculocutaneous albinism II | *Oca2* | 18431 | -1.08 | -1.14 | -1.54 | -1.49 | -1.23 |
| UDP-N-acteylglucosamine pyrophosphorylase 1-like 1 | *Uap1l1* | 227620 | -1.06 | -1.28 | -1.54 | -1.07 | 1.03 |
| basic helix-loop-helix domain containing, class B, 3 | *Bhlhb3* | 79362 | -1.07 | -1.20 | -1.53 | -1.42 | -1.04 |
| guanylate cyclase activator 1B (retina) | *Guca1b* | 107477 | -1.11 | -1.15 | -1.52 | -1.24 | -1.61 |
| EPH receptor A3 | *Epha3* | 13837 | -1.04 | 1.02 | -1.52 | -1.22 | -1.11 |
| indolethylamine N-methyltransferase | *Inmt* | 21743 | 1.13 | -1.42 | -1.52 | -2.16 | -1.89 |
| glioma-associated oncogene homolog 1 (zinc finger protein) | *Gli1* | 14632 | -1.13 | -1.18 | -1.51 | -1.74 | -1.48 |
| hyaluronan synthase 2 | *Has2* | 15117 | -1.05 | 1.00 | -1.51 | -1.48 | -1.41 |
| protein kinase C, theta | *Prkcq* | 18761 | -1.05 | -1.33 | -1.50 | -1.45 | -1.09 |
| syntrophin, beta 1 (dystrophin-associated protein A1, 59kDa, basic component 1) | *Sntb1* | 20649 | -1.13 | -1.29 | -1.50 | -1.17 | 1.02 |
| crystallin, mu | *Crym* | 12971 | 1.06 | -1.03 | -1.47 | -1.44 | -1.62 |
| hyaluronan and proteoglycan link protein 1 | *Hapln1* | 12950 | -1.12 | -1.07 | -1.47 | 1.03 | 1.15 |
| keratin 18 | *Krt18* | 16668 | 1.04 | -1.25 | -1.47 | -1.41 | -1.22 |
| microphthalmia-associated transcription factor | *Mitf* | 17342 | -1.03 | -1.31 | -1.43 | -1.19 | 1.13 |
