# Supplementary material for: A Regulatory Loop Involving PAX6, MITF, and WNT Signaling Controls Retinal Pigment Epithelium Development
Source: PLoS Genet. 2012 Jul 5;8(7):e1002757. doi: 10.1371/journal.pgen.1002757 (PMC3390378; doi:10.1371/journal.pgen.1002757)
Supplement: Table S3 — List of used primers. List of primers used to generate Mitfmi-ΔD/Mitfmi-ΔD and Tfec overexpressing transgenic mice, to perform genotyping, RT-PCR, and ChIP assays, and to construct reporter plasmids is listed. (DOCX) [file pgen.1002757.s011.docx]

| **Table S3: list of used primers** | | |
| --- | --- | --- |
| # | **Primers for generating *Mitf^mi-^Δ^D^/Mitf^mi-^Δ^D^* mice** | **Specific purpose** |
| 1  2 | 5’- tacccgcggccgctcctgcatgtagaaagaacc -3' (for)  5’- ggggcaagcttgatatgtatgaaacccaagagg -3’ (rev) | cloning of *D-Mitf* locus -upstream flank |
| 3  4 | 5’- ctaacaagctttagagagctgatagctatctagac -3’ (for)  5’- tctatactagtcataatgcacacaattagcc -3’ (rev) | cloning of *D-Mitf* locus -downstream flank |
| 5  6 | 5’- taaaaccgcggttctggtatcgatgattccatgaag -3’ (for)  5’- catgtggatccactggctggagtactgggag -3’ (rev) | upstream flank for *D-Mitf* promoter/exon, genotyping *Mitf^mi-^Δ^D^* allele |
| 7  8 | 5’- gcaaagaattcacgctgcttggtcagtgttcc -3’ (for)  5’- gcaatggtaccaatagacactaactaaagaaaatgacagg -3’ (rev) | downstream flank for *D-Mitf* promoter/exon, genotyping *Mitf^mi-^Δ^D^* allele |
| 9  10 | 5’- ggtttacctcctcttactgtcttgc -3’ (for)  5’- agataactaagaagctatgcctctcc -3’ (rev) | upstream probe for Southern hybridization |
| 11  12 | 5’- atagttttaagtagcagggcaggtgc -3’ (for)  5’- gaggctgtatctcatgaattctggc -3’ (rev) | downstream probe for Southern hybridization |
| 13  14 | 5’- ctttagagctctttattcagctgtcaattagctgagc -3’ (for)  5’- tattcggatcctgtcagggcccaacattctcg -3’ (rev) | genotyping primers – wt *D-Mitf* allele |
| 15  16 | 5’- caccatgatattcggcaagcaggc -3’ (for)  5’- tgactgggcacaacagacaatcgg -3’ (rev) | genotyping primer –Neomycin |
|  | **Generating RPE-specific *Tfec* overexpressing transgenic mouse** |  |
| 17  18 | 5’- gctcggatccatgacctttgactgtcgggtatgc -3’ (for)  5’- gatatctagacaactcatcaccatcctctgagc -3’ (rev) | Tfec-cDNA cloning |
| 19  20 | 5’- agctcgtcgacatgacctttgactgtcgggtatgc -3’ (for)  5’- ctgttgtcgacagtcataggccgaatagcctc -3’ (rev) | Tfec-V5 cDNA cloning |
|  | **Other genotyping primers** |  |
| 21  22 | 5’- tgccagcaacaggaaggagg -3’ (for)  5’- atggaacctgatgtgaaggagg -3’ (rev) | genotyping primers – *Pax6^Sey-Neu^* allele. Cut PCR with HincII – wt 1100 bp;  mutant 950 bp, 150bp |
| 23  24 | 5’- caaacaggtttaaagacattg -3’ (for)  5’- acagaggtgcttgtacagagt -3’ (rev) | genotyping primers – *Pax6^Yac^* allele |
| 25  26 | 5’- gcatcgagctgggcaataagcg -3’ (for)  5’- gacaccacaccagctggtaatggtagcg -3’ (rev) | genotyping primers – *Mitf^mi^* allele |
| 27  28 | 5’- gctctcctgtagcccaagatagg -3’ (for)  5’- aaggtggaggtatgcctgagagg -3’ (rev) | genotyping primers – *Mitf^mi-rw^* allele |
| 29  30 | 5’- ggatgaggttcgcaagaacc -3’ (for)  5’- ccatgagtgaacgaacctgg -3’ (rev) | genotyping primers – *Cre recombinase* |
|  | **RT-PCR primers** |  |
| 31  32 | 5’- acaggtatggttttctaatcgaagg -3’ (for)  5’- aacatggagccagatgtgaaggagg -3’ (rev) | *Pax6* – both human and mouse alleles |
| 33  34 | 5’- ccacacctgtctcctccttc -3’ (for)  5’- gaggggtgtaggtatcataactcc -3’ (rev) | *Pax6* – only WT mouse allele |
| 35  36 | 5’- cggcagaagatcgtagagctagc -3’ (for)  5’- ccagtataaacgggagtgcccttc -3’ (rev) | *Pax6* – 5a+/5a- isoforms |
| 37  38 | 5’- aagctgaattcctatgacatcacgcatcttgc -3’ (for)  5’- gcagacccacctggaaaacccc -3’ (rev) | *Mitf-Pan* (exon B-exon 2a) |
| 39  40 | 5’- gcggatttcgaagtcggggagg -3’ (for)  5’- ccagccataaacgtcagcgtgc -3’ (rev) | *A-Mitf* |
| 41  42 | 5’- tctcgccgtgtctctgggcatc -3’ (for)  5’- ccagccataaacgtcagcgtgc -3’ (rev) | *J-Mitf* |
| 43  44 | 5’- gggcttgcagaacaccttaaagg -3’ (for)  5’- ccagccataaacgtcagcgtgc -3’ (rev) | *H-Mitf* |
| 45  46 | 5’- gttgggacctgacaggctctgaatacag -3’ (for)  5’- ccagccataaacgtcagcgtgc -3’ (rev) | *D-Mitf* |
| 47  48 | 5’- ggaaatgctagaatacagtcactacc -3’ (for)  5’- catgcacgacgctcgagagtgc -3’ (rev) | *M-Mitf* |
| 49  50 | 5’- tcttatttgacccctatgcatcacc -3’ (for)  5’- cagtcagcattgaagttaagcttcc -3’ (rev) | *Otx2* |
| 51  52 | 5’- caggaagaactgagggagaacagc -3’ (for)  5’- ccatgtcctccagctgcggtgg -3’ (rev) | *Vsx2* |
| 53  54 | 5’- cgccttcgagaagtcccactacc -3’ (for)  5’- gagcggctgaaggagaggagg -3’ (rev) | *Rax* |
| 55  56 | 5’- taaggagtctcacggcaagctgc -3’ (for)  5’- gcgccagttcgcgtttcttgctgg -3’ (rev) | *Six3* |
| 57  58 | 5’- accgcgagctttatcatatcctgg -3’ (for)  5’- ggtaccactctcgaagcaggtgc -3’ (rev) | *Six6* |
| 59  60 | 5’- gagggagcagctgctgagacac -3’ (for)  5’- gctccctccctgcaatactt -3’ (rev) | *Usf1* |
| 61  62 | 5’- cacagctgagagggaaatcgtgc -3’ (for)  5’- gatcttgatcttcatggtgctagg -3’ (rev) | β-actin |
| 63  64 | 5’- ccgagacagtgagtgctgtgg -3’ (for)  5’- catggcagagctctccctcc -3’ (rev) | Dkk3 |
| 65  66 | 5’- atgtctccaactgcttcctccg -3’ (for)  5’- aaggtacagtcttcctccgag -3’ (rev) | Fgf15 |
|  | **Vectors for luciferase reporter assays** |  |
| 67  68 | 5’- tgtctgagctccttgggaagatcaattattcttgg -3’ (for)  5’- ttgattaagcttgtcatcatatttgtttaaagcacag -3’ (rev)  (700 bp of minimal promoter and proximal enhancer region containing PAX6 and MITF binding sites) | pGL4-Tfec-enh-prom-luciferase |
| 69  70  71  72 | 5’- ttagaggtaccaaggtgagctcaagaaggctg -3’ (for)  5’- ccaggaagctttccacttgacacgcttatgg -3’ (rev)  5’- aagcaggtacctttgcttgaaggactggg -3’ (for)  5’- tcaaggagctctgtttactcacaggagcgtac -3’ (rev)  (572 bp of distal enhancer region, -6372 to -5778, containing PAX6 and MITF binding sites directly fused to 665 bp of minimal promoter) | pGL4-Dkk3-enh-prom-luciferase |
| 73  74 | 5’- tccctggtaccccacatttatcacg -3’ (for)  5’- tcctgaagcttgttacaaaggtggcgcg -3’ (rev)  (1450 bp of minimal promoter + distal enhancer region containing PAX6 and MITF binding sites) | pGL4-Fgf15-enh-prom-luciferase |
| 75  76 | 5’- atcgaggatccatgcagaacagtcacagcggagtg -3’ (for)  5’- tcgattctagactgtaatcttggccagtattgag -3’ (rev) | pcDNA3.1-Pax6-V5-HisA |
| 77 | Bharti et al., 2008 | pcDNA3.1-D-Mitf-V5-HisA |
| 78 | see above | pcDNA3.1-Tfec-V5-HisA |
|  | **ChIP assays** |  |
| 79  80 | 5’- gcaataacggcatcaatgagagg -3’ (for)  5’- ttaaggaagaagaaatgacacagc -3’ (rev) | *Tfec promoter*  – region 1 |
| 81  82 | 5’- actgaaagtgggcaaggacag -3’ (for)  5’- tgccccataataaacatttttcc -3’ (rev) | *Tfec promoter*  – region 2 |
| 83  84 | 5’- taaaggctaggaataaggtcctatgg -3’ (for)  5’- atggaaattccaactgcccttgg -3’ (rev) | *Dkk3 promoter*  – region 1 |
| 85  86 | 5’- gggttctataataataactactcacc -3’ (for)  5’- gagaacctggaggctggttgc-3’ (rev) | *Dkk3 promoter*  – region 2 |
| 87  88 | 5’- tcctcctctgccagatctgg-3’ (for)  5’- -cgcggccttatatacgtttcg -3’ (rev) | *Fgf15 promoter*  – region 1 |

Restriction enzyme sites are underlined
