# Supplementary material for: A Regulatory Loop Involving PAX6, MITF, and WNT Signaling Controls Retinal Pigment Epithelium Development
Source: PLoS Genet. 2012 Jul 5;8(7):e1002757. doi: 10.1371/journal.pgen.1002757 (PMC3390378; doi:10.1371/journal.pgen.1002757)
Supplement: Table S4 — List of used in situ hybridization probes. In situ probes used in this study are listed. (DOCX) [file pgen.1002757.s012.docx]

| **Table S4: list of used in situ hybridization probes** | | |
| --- | --- | --- |
| **#** | **Probes** | **Source** |
| 1 | Vsx2 | Open Biosystems, Huntsville, Al |
| 2 | Rax | Open Biosystems, Huntsville, Al |
| 3 | Six6 | Bharti et al., 2010 |
| 4 | Otx2 | Gift Stefano Bertuzzi |
| 5 | Six3 | Gift Stefano Bertuzzi |
| 6 | Tfec | Open Biosystems, Huntsville, Al |
| 7 | Crx | Gift Stefano Bertuzzi |
| 8 | Math3 | Gift Stefano Bertuzzi |
| 9 | Dkk3 | Open Biosystems, Huntsville, Al |
| 10 | Fgf15 | Open Biosystems, Huntsville, Al |
| 11 | Connexin 43 | Open Biosystems, Huntsville, Al |
| 12 | Tbx5 | Gift Stefano Bertuzzi |
| 13 | Vax2 | Gift Stefano Bertuzzi |
