# Supplementary material for: A Regulatory Loop Involving PAX6, MITF, and WNT Signaling Controls Retinal Pigment Epithelium Development
Source: PLoS Genet. 2012 Jul 5;8(7):e1002757. doi: 10.1371/journal.pgen.1002757 (PMC3390378; doi:10.1371/journal.pgen.1002757)
Supplement: Table S5 — List of used antibodies. Antibodies used in this study, their commercial source, species and dilution is listed. (DOCX) [file pgen.1002757.s013.docx]

| **Table S5: list of used antibodies** | | | | |
| --- | --- | --- | --- | --- |
| **#** | **Antibodies** | **Source** | **Species** | **Dilution** |
| 1 | anti-PAX6 | Convance, Princeton, NJ | Rabbit | 1:200 |
| 2 | anti-MITF (LH8) | Polyclonal, (Opdecamp et al., Development 124, 2377-2386, 1997) | Rabbit | 1:200 |
| 3 | anti-MITF (6A5) | Monoclonal (Bharti et al., Development 135, 1169-1178, 2008) | Mouse | 1:200 |
| 4 | anti-CD138 | BDbioscience, San Jose, CA | Rat | 1:500 |
| 5 | anti-TYROSINASE | Gift, Dr. V. Hearing NCI/NIH | Rabbit | 1:500 |
| 6 | anti-phosphohistone H3 | Upstate Biotechnology, Billerica, MA | Rabbit | 1:100 |
| 7 | anti-TUJ1 | Convance, Princeton, NJ | Mouse | 1:1000 |
| 8 | anti-SOX2 | Convance, Princeton, NJ | Rabbit | 1:500 |
| 9 | AlexaFluor 488  Phalloidin | Invitrogen, Eugene, OR | N/A | 1:100 |
| 10 | anti-V5 | Invitrogen, Eugene, OR | Mouse | 1:2000 |
| 11 | anti-ISL1 | Hybridoma Bank, University of Iowa | Mouse | 1:500 |
| 12 | anti-NF160 | Sigma | Mouse | 1:500 |
| 13 | anti-SYNTAXIN | Sigma | Mouse | 1:500 |
| 14 | anti-VC1.1 | Sigma | Mouse | 1:500 |
| 15 | anti-VSX2 | Abcam, Cambridge, MA | Sheep | 1:200 |
| 16 | anti-βGAL | MP Biomedicals, Solon, OH | Rabbit | 1:200 |
| 17 | anti-rabbit-488 | Invitrogen, Eugene, OR | Goat | 1:1000 |
| 18 | anti-acetyl Histone H3 | Upstate Biotechnology, Billerica, MA | Rabbit | 1:200 |
| 19 | anti-dimethyl Histone H3 | Upstate Biotechnology, Billerica, MA | Rabbit | 1:200 |
| 20 | anti-rabbit-564 | Invitrogen, Eugene, OR | Goat | 1:1000 |
| 21 | anti-mouse-488 | Invitrogen, Eugene, OR | Goat | 1:1000 |
| 22 | anti-mouse-564 | Invitrogen, Eugene, OR | Goat | 1:1000 |
| 23 | anti-rat-564 | Invitrogen, Eugene, OR | Goat | 1:1000 |
